# Serum Cytokines Predict Neurological Damage in Genetically Diverse Mouse Models

**DOI:** 10.3390/cells11132044

**Published:** 2022-06-28

**Authors:** Aracely A. Pérez Gómez, Moumita Karmakar, Raymond J. Carroll, Koedi S. Lawley, Katia Amstalden, Colin R. Young, David W. Threadgill, C. Jane Welsh, Candice Brinkmeyer-Langford

**Affiliations:** 1 Interdisciplinary Faculty of Toxicology, College of Veterinary Medicine and Biomedical Sciences, Texas A & M University, College Station, TX 77843, USA; dwthreadgill@tamu.edu; 2 Department of Veterinary Integrative Biosciences, College of Veterinary Medicine and Biomedical Sciences, Texas A & M University, College Station, TX 77843, USA; koedilawley@tamu.edu (K.S.L.); kamstalden@cvm.tamu.edu (K.A.); cyoung@cvm.tamu.edu (C.R.Y.); jwelsh@cvm.tamu.edu (C.J.W.); 3 Department of Statistics, College of Science, Texas A & M University, College Station, TX 77843, USA; mkarmakar@stat.tamu.edu (M.K.); carroll@stat.tamu.edu (R.J.C.); 4 Department of Molecular and Cellular Medicine, Texas A & M Health Science Center, Texas A & M University, College Station, TX 77843, USA

**Keywords:** TMEV, cytokine, acute, infection, virus, immune response-profile, neurological, disease

## Abstract

Viral infections contribute to neurological and immunological dysfunction driven by complex genetic networks. Theiler’s murine encephalomyelitis virus (TMEV) causes neurological dysfunction in mice and can model human outcomes to viral infections. Here, we used genetically distinct mice from five Collaborative Cross mouse strains and C57BL/6J to demonstrate how TMEV-induced immune responses in serum may predict neurological outcomes in acute infection. To test the hypothesis that serum cytokine levels can provide biomarkers for phenotypic outcomes of acute disease, we compared cytokine levels at pre-injection, 4 days post-injection (d.p.i.), and 14 d.p.i. Each strain produced unique baseline cytokine levels and had distinct immune responses to the injection procedure itself. Thus, we eliminated the baseline responses to the injection procedure itself and identified cytokines and chemokines induced specifically by TMEV infection. Then, we identified strain-specific longitudinal cytokine profiles in serum during acute disease. Using stepwise regression analysis, we identified serum immune markers predictive for TMEV-induced neurological phenotypes of the acute phase, e.g., IL-9 for limb paralysis; and TNF-α, IL-1β, and MIP-1β for limb weakness. These findings indicate how temporal differences in immune responses are influenced by host genetic background and demonstrate the potential of serum biomarkers to track the neurological effects of viral infection.

## 1. Introduction

Viruses are abundant, ubiquitous in nature, and may be deleterious to human health. Prior viral infections, including Epstein-Barr virus (EBV) [1,2], Herpes Simplex virus (HSV) [3,4], Human immunodeficiency virus (HIV) [5,6], poliovirus [7,8], Zika virus [9,10], and many others have been associated with subsequent neurological damage [11]. Comorbidities and predisposing genetic risk factors for neurological and immunological dysfunction often vary among individuals in natural populations. As a result, any single viral infection may contribute to a spectrum of neurological and immunological outcomes, including diseases such as amyotrophic lateral sclerosis (ALS) [12,13], epilepsy [14,15], multiple sclerosis (MS) [16,17], and Parkinson’s disease (PD) [18,19].

Theiler’s murine encephalomyelitis virus (TMEV) is a naturally occurring neurotropic, single-stranded RNA murine virus, often used to model human neurological damage associated with viral infections, e.g., epilepsy and MS [20,21]. While studies in inbred mouse models have established different pathologies after TMEV infection, including seizures and demyelinating disease [22,23], a consistent finding is the variability of immune responses detected among infected models [24,25].

Research with TMEV infected models suggests that neurological damage can be attributed to improper induction and regulation of the immune response during the acute phase of infection (the first two weeks post-infection) [26]. In vitro experiments show that TMEV infects the following cells lytically: neurons, oligodendrocytes [27], and endothelial cells [28], resulting in cell lysis and viral persistence in macrophages and astrocytes [23,27]. Similarly, in vivo TMEV infection is evident in neurons during early disease and later in astrocytes, oligodendrocytes, microglia, and macrophages [29,30]. This infection of resident central nervous system (CNS) cells activates both innate and adaptive immune responses to induce a rapid pro-inflammatory response needed to restrict viral replication. This response to TMEV infection generally includes the initiation of leukocyte extravasation, neutrophil production, and macrophage infiltration for the removal of virally-infected cells by the release of pro-inflammatory cytokines and chemokines such as IL-1, IL-6, and TNF-α, [31]. In seizure-susceptible strains, such as C57BL/6 (B6) mice, this response effectively clears the virus but results in severe bystander damage that promotes epilepsy. In contrast, mouse strains susceptible to TMEV-induced demyelination, such as SJL/J, fail to clear the virus due to a dampened pro-inflammatory response and ensure debilitating CNS pathology in the chronic phase, resembling MS [32,33].

Previous research has exploited the phenotypic reproducibility and utility of inbred mice, such as C57BL/6J and SJL/J, to produce in-depth analyses of host-pathogen interactions. However, the limited genetic diversity in common inbred strains underrepresents the phenotypic outcomes in heterogeneous populations comparable to humans. The Collaborative Cross (CC) resource, composed of many recombinant and reproducible inbred mouse lines, overcomes this issue [34,35]. The CC model was established by cross-breeding eight genetically diverse founder strains (A/J, C57BL/6J, 129S1/SvImJ, NOD/LtJ, NZO/HlLtJ, CAST/EiJ, PWK/PhJ, and WSB/EiJ) in a combinatorial funnel scheme until achieving maximum recombination [36]. As a result, the expanded pool of genetic variation rendered each CC mouse strain a genetically unique “individual”—a model for human genetic heterogeneity.

In the present study, immune responses from CC mice representing different TMEV-induced phenotypes, reminiscent of human neurological diseases, were evaluated during the acute phase of infection. We hypothesized that different clinical outcomes induced by TMEV infection are associated with unique cytokine and chemokine profiles in serum. To test our hypothesis, we selected a pre-injection time point and two time points within the acute phase of infection to characterize longitudinal cytokine and chemokine profiles in serum for each strain. Our findings provide an understanding of temporal changes in the immune response to neurotropic viral infections and reveal those changes which contribute to neurological disease. Furthermore, we associated profiles of pre-injection (baseline) levels of cytokines and chemokines as predictors of relative risk for developing TMEV-induced neurological dysfunction. Identifying the time sequence of the immunological response may inform the development of appropriate models of disease and immunotherapies for humans susceptible to virus-induced neurological disorders such as ALS, MS, and PD. Importantly, these predictive biomarkers can be evaluated in serum, offering a valuable approach for diagnostic and prognostic testing in patients.

## 2. Materials and Methods

### 2.1. Ethics Statement

All animal care protocols were approved by Texas A&M University Laboratory Animal Care and Use Committee (AUP 2020-0065, approved 21 May 2020) and complied with NIH Guidelines for Care and Use of Laboratory Animals. Mice were group-housed, and all testing was performed during the light phase.

### 2.2. Experimental Design

**Figure 1:**
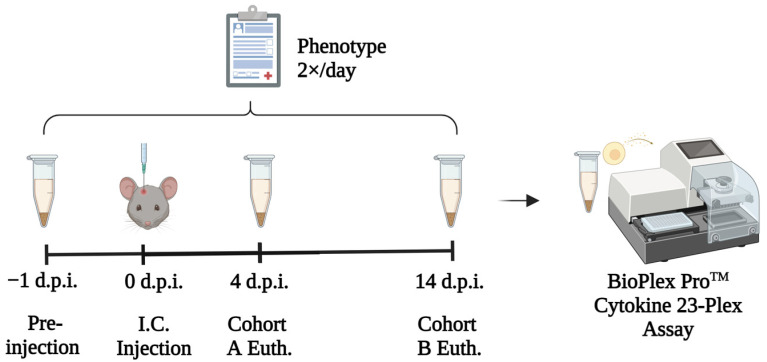
Schematic of experimental design used for this study. “Euth: Euthanasia”.

In the present study, we hypothesized that different TMEV-induced phenotypic outcomes are associated with unique cytokine and chemokine profiles in serum. To test our hypothesis, we established the experimental design described below (Figure 1).

### 2.3. Mouse Management

The strains and numbers of mice in the study depended on the reproductive success of our in-house breeding system. The mice were maintained under 14-h light and 10-h dark cycles with ad libitum food and water. Mice were specific pathogen-free and housed in polycarbonate cages with filtered lids, with a maximum of four adult mice per cage and cage cleanouts twice per week. The facility where the mice were housed adheres to federal regulations and guidelines (Animal Welfare Act; Guide for the Care and Use of Laboratory Animals; Guide for the Care and Use of Agricultural Animals in Agricultural Research and Teaching) regarding animal housing, hygiene, and care.

Female and male mice of five Collaborative Cross (CC) strains and C57BL/6J (B6) were randomly assigned to two study cohorts– A (studied up to four days post-injection [d.p.i.]) and B (up to 14 d.p.i.). Mice from each strain were then randomly sorted into exposure groups—PBS-injected or TMEV-infected (Table 1). Mice at four weeks of age were anesthetized by isoflurane inhalation (MWI, Meridian, ID, USA) and inoculated into the right mid-parietal cortex at a depth of ~1.5 mm with 20 μL of 1 × Phosphate Buffer Solution (PBS) (PBS-injected/control mice), or with 5.0 × 10^4^ plaque-forming units (PFU) of BeAn strain of TMEV (TMEV-injected/infected mice) (American Type Culture Collection [ATCC] VR 995, Manassas, VA, USA), as previously used in [25,37,38,39,40]. Mice were housed separately by exposure groups.

### 2.4. Qualitative Neurological Phenotyping

Mice were weighed and phenotypically evaluated before and after injection until either 4 d.p.i. (Cohort A) or 14 d.p.i. (Cohort B). Phenotypes, such as limb clasping and delay in righting reflex, were evaluated and scored as described previously [38]. Control and infected mice were scored for all phenotypes, with the findings from PBS-injected mice serving as a control for scoring the infected mice. Therefore, behaviors such as ruffling (piloerection) were recorded only if seen in infected but not control mice of the same gender and strain [41,42,43,44]. Limb clasping was evaluated and scored as previously described [38,45]; righting reflex scores were determined by how long each mouse took to right itself from a prone position, as described [38]. Clinical signs of limb weakness and paralysis were observed and scored on a scale of 0–4, with a score of 0 given to mice having normal stride and no signs of weakness, and a score of 4 representing the total loss of limb mobility characterized by lack of grip function and flaccid limb extension [25].

### 2.5. Serum Collection and Euthanasia

Blood was collected before injection (at four weeks of age) from the submandibular vein via puncture with a 25-gauge needle at a depth of ~1 mm. At the end of the study, mice were euthanized at 4 d.p.i. or 14 d.p.i. by intraperitoneal (IP) injection of Beuthansia 150 mg/kg (Merck & Co., Kenilworth, NJ, USA) as described [44]. Then, blood was collected from the right axillary vessel, and mice were perfused with a 1 × PBS solution through the left ventricle. Collected blood was refrigerated at 4 °C for an hour and then centrifuged at 2000× *g* rpm. Sera collected from the supernatants were stored at −20 °C for further analysis.

### 2.6. Cytokine and Chemokine Assays

We used the serum collected before (baseline) and after injection (at 4 d.p.i. and 14 d.p.i.) to evaluate TMEV-induced immune responses. Immune response proteins were measured with Bio-Plex Pro^TM^ Mouse Cytokine 23-plex Assay kit (Bio-Rad, Hercules, CA, USA) to determine concentration levels of 23 cytokines and chemokines (IL-1α, IL-1β, IL-2, IL-3, IL-4, IL-5, IL-6, IL-9, IL-10, IL-12p40, IL-12p70, IL-13, IL-17α, IFN-γ, CCL11 [Eotaxin], G-CSF, GM-CSF, CXCL1 [KC {keratinocyte-derived chemokine}], CCL2 [MCP-1 {Monocyte Chemotactic Peptide 1}]), CCL3 [MIP-1α {Macrophage Inflammatory Protein 1α}], CCL4 [MIP-1β], CCL5 [RANTES], and TNF-α). Data were processed and analyzed using the Bio-Plex Manager software program (Bio-Rad version 4.1.1, Hercules, CA, USA).

### 2.7. Statistics

GraphPad Prism version 9.3.1 for Mac (GraphPad Software, San Diego, CA, USA) was used to perform nonparametric Mann-Whitney U tests for comparing ﻿cytokine and chemokine levels among control and TMEV-infected mice within the same CC strain. Prism was also used to perform 2-way ANOVA for comparing gender-specific differences among control and TMEV-infected mice of each gender. All reported *p* values are based on two-tailed statistical tests with a significance level of 0.05.

**Figure 2:**
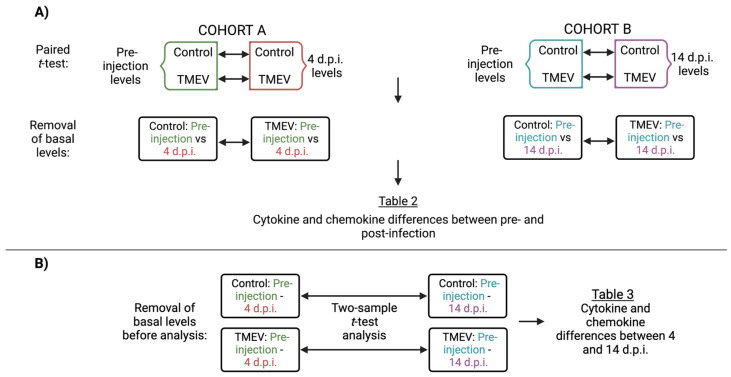
Step-by-step analyses revealed cytokines and chemokines with changes attributable solely to immune responses to the i.c. injection for each strain. (**A**) Paired *t*-test analysis to determine differences between pre-and post-injection responses (pre-injection vs. 4 d.p.i. and pre-injection vs. 14 d.p.i.). (**B**) Two-sample *t*-test analysis to identify differences between the 4 and 14 d.p.i. period after normalizing both post-injection time points with their respective pre-injection values (Created with BioRender.com).

Paired, two-sample *t*-tests and stepwise regression statistical analyses were performed using R software (version 4.0.3, R Core Team, Vienna, Austria) Paired *t*-tests revealed statistically significant temporal production of cytokines and chemokines concerning residual responses from the intracranial (i.c.) injection and to TMEV infection (Figure 2). The stepwise regression model allows for the identification of a list of plausible explanatory variables that have causal effects on the dependent variable. In our study, this method was implemented to identify predictive biomarkers using pre-injection cytokine serum levels to the viral-induced neurological symptoms. The algorithm performs stepwise regression based on a nested model test for the inclusion and exclusion of a predictor. The stepwise regression procedure involves a forward selection mechanism that starts with the intercept-only model and proceeds according to the optimal stopping criterion to choose the final model. Following [25,46], inclusion and exclusion of variables were controlled by alpha to enter and alpha to leave parameters, in which both were set to 0.05. Additional details about these statistical analyses are available in Supplementary Statistical Methods.

## 3. Results

### 3.1. TMEV-Induced Phenotypes

We focused on five CC strains we had found to represent maximum phenotypic divergence (e.g., mild to severe disease) based on their phenotypic responses to TMEV during the chronic phase of infection, described in detail [25,38,39,47]. In the current study, TMEV significantly induced various clinical symptoms such as a decreased righting response, hunching, and limb paralysis in those strains most severely affected by TMEV, allowing for strain categorization based on acute phenotypes. No neurological symptoms associated with TMEV infection occurred in any PBS-injected mice.

**Figure 3:**
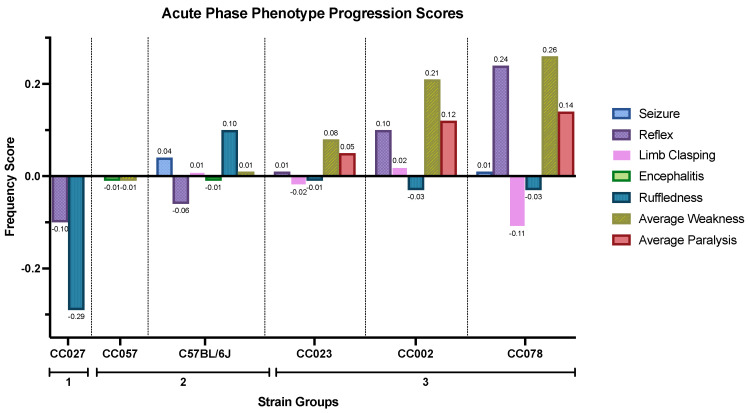
Cumulative progression scores of neurological phenotypes varied by strain. The difference between the cumulative scores for all neurological phenotypes (listed in the legend) between 4 and 14 d.p.i. summarized how each strain fared following TMEV infection. These scores are presented above or below each phenotype column. The y-axis shows the cumulative frequency score for each strain; these scores reflect the relative frequency difference of observation for each listed symptom over time. Positive frequency scores indicate symptoms worsened (more frequent) from 4 to 14 d.p.i.; negative scores indicate symptoms improved (less frequent) from 4 to 14 d.p.i. Each CC strain is listed along the x-axis by increasing the cumulative frequency score. These strains were classified based on whether their symptoms showed overall improvement (group 1), a balance between worsening and improving symptoms (group 2), or showed overall worsening (group 3). Not shown are phenotypes with scores of 0.

Frequency scores for each phenotype were calculated as we have described previously [38]. Essentially, these scores reflect how often each phenotype was observed and to what extent (e.g., how many limbs were concurrently affected and the degree of function lost relative to PBS-injected mice of the same gender and strain). Cumulative frequency scores are the total values for all frequency scores at a given time point and provide a snapshot of the degree to which an individual mouse presented TMEV-induced phenotypes at a given time (Supplementary Figure S1). We focused on disease progression, which we defined as the difference of cumulative frequency scores between two time points; calculated for each strain by obtaining the differences between scores at 4 and 14 d.p.i. Based on the degree to which cumulative frequency scores increased or decreased for each strain, we classified the strains into three categories (Figure 3).

Strains CC002, CC023, and CC078 were the most susceptible to TMEV acute phase symptoms as demonstrated by the progression of clinical signs from 4 d.p.i. to 14 d.p.i. and were then assigned to group 3. Although B6 mice developed seizures, this strain has been consistently described as “resistant” to demyelination and TMEV persistence [20,48,49], so we grouped B6 mice in the less-affected group 2. We previously defined CC057 as TMEV-susceptible based on phenotypic progression over 90 d.p.i. [38]. However, the focus of the current study was the acute phase of infection, and CC057 mice did not exhibit progressive TMEV-induced phenotypes during this stage. Therefore, we classified CC057 as group 2. Any phenotypes experienced by CC027 mice were restored to pre-injection levels by the end of the acute phase; CC027 mice were scored as group 1.

### 3.2. Identification of Immune Responses Influenced by Intracranial Injection

Cytokine and chemokine production is essential to avoid the detrimental effects of acute-phase viral infection. However, levels of and interactions between serum and CNS cytokines and chemokines may initiate a cascade of events leading to irreversible damage. While, typically, immune responses are measured in CNS tissue, we focus on profiling the immune responses in sera to further characterize the overall impact TMEV has in the periphery. A total of 23 cytokines and chemokines were measured in serum collected before and after i.c. injection. We performed paired *t*-tests between pre-injection and post-injection (pre-injection vs. 4 d.p.i. and pre-injection vs. 14 d.p.i.) measurements as described (Figure 2A). Thus, we accounted for the baseline responses to the injection procedure itself and identified cytokines and chemokines induced specifically by the i.c. injection (Supplementary Table S1).

Our analysis revealed distinct changes in the production of many cytokines and chemokines influenced by the i.c. injection throughout different post-injection time points, 4 and 14 d.p.i. (Table 2). Accordingly, these changes were attributed solely to the injury induced by the injection itself. Typically, after a brain injury, such as the i.c. injection procedure, inflammatory responses would be activated, resulting in the production of inflammatory agents (e.g., IL-1α, IL-1β, TNF-α, IFN-γ, IL-7, RANTES, and others) [50]. Therefore, it was not surprising to find changes in the levels of these proteins. However, TNF-α, which has been previously associated with brain injury [51], was not influenced by the injection procedure (TNF-α column; Table 2). Similarly, the pre-injection procedure affected anti-inflammatory IL-4 responses in CC078 mice, levels of pro-inflammatory IL-1α only in CC057 mice, and granulocyte stimulator GM-CSF in B6 mice. Therefore, it would seem the effects of the i.c. injection procedure does not resemble those of a traumatic brain injury. Interestingly, IL-9 was the only cytokine produced at both time points in all strains within the same phenotypic group, in this case, Group 2 (B6 and CC057 mice). The rest of the cytokines and chemokines produced in response to the i.c. injection have the potential to aggravate further or resolve TMEV-induced phenotypic outcomes.

### 3.3. Characterization of Immune Responses Elicited Solely by TMEV Infection

We sought to identify differences in cyto/chemokines between PBS-injected and TMEV-infected strains across the acute phase of infection. In Table 2, we summarized the strain-specific impacts of the i.c. injection on the immune profiles. We next focused on the effects of TMEV infection, specifically on cyto/chemokine levels. Cytokines and chemokines produced in response to the i.c injection were normalized via paired difference analysis by subtracting pre-injection measurement levels from measurements taken at 4 and 14 d.p.i. Then, we performed a two-sample *t*-test on control and infected mice, as described in Figure 2B, to identify significant variations between cytokines and chemokines produced between the 4 and 14 d.p.i time points (Table 3). We anticipated strain-specific differences in the cytokines and chemokines produced by control and infected mice, which allowed for identifying responses solely induced by TMEV infection. Overall, those cytokines that vary throughout the acute phase of infection are characteristic of inducing and maintaining a pro-inflammatory environment (e.g., GM-CSF, IL-17α, MIP-1α, and RANTES).

Typically, cytokines belonging to the pro-inflammatory triad (IL-1, IL-6, and TNF-α) are induced in response to pathogens. However, we observed that only IL-6 was identified throughout the acute phase in this analysis, while IL-1 and TNF-α were not produced in response to TMEV infection. High levels of TNF-α may lead to cytotoxicity and the induction of harmful molecular mechanisms known to cause CNS damage. However, we found TNF-α levels at 4 and 14 d.p.i. to be consistent with pre-injection levels, meeting the criteria for inclusion in Table 2 but not in Table 3—suggesting TNF-α did not play a significant role in acute phase phenotypes for any strain but rather was induced in response to the i.c. injection. Those cytokines and chemokines identified in both sets of analyses suggest an augmentation might have occurred throughout the acute phase. Overall, the cytokines and chemokines produced during the acute infection were not in the pro-inflammatory triad but instead consisted of other cytokines known to promote an inflammatory environment, such as IL-12p40, IL-17α, RANTES, and IFN-γ.

### 3.4. Cytokine and Chemokine Influence on TMEV-Induced Phenotypes

Next, we quantified 23 different cytokine and chemokine serum levels at individual time points to capture a “snapshot” of the differences between control and infected mice, including any residual responses to the injection. Similar to findings from our previous study of chronic phase (90 d.p.i.) cytokine and chemokine levels [25], most cytokine and chemokine levels in infected mice mirrored those measured in PBS-injected mice (Supplementary Figure S2A–D).

**Figure 4:**
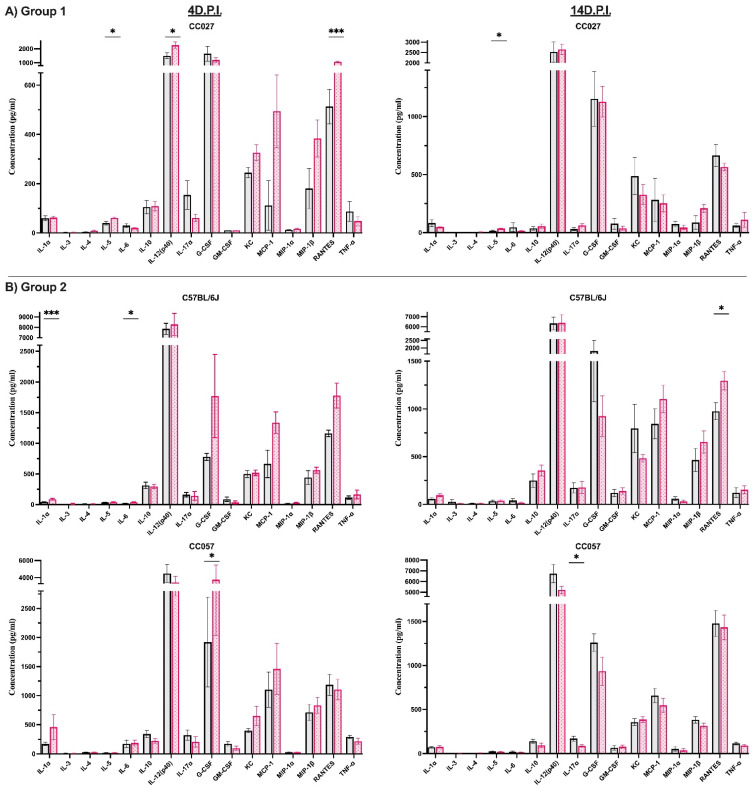

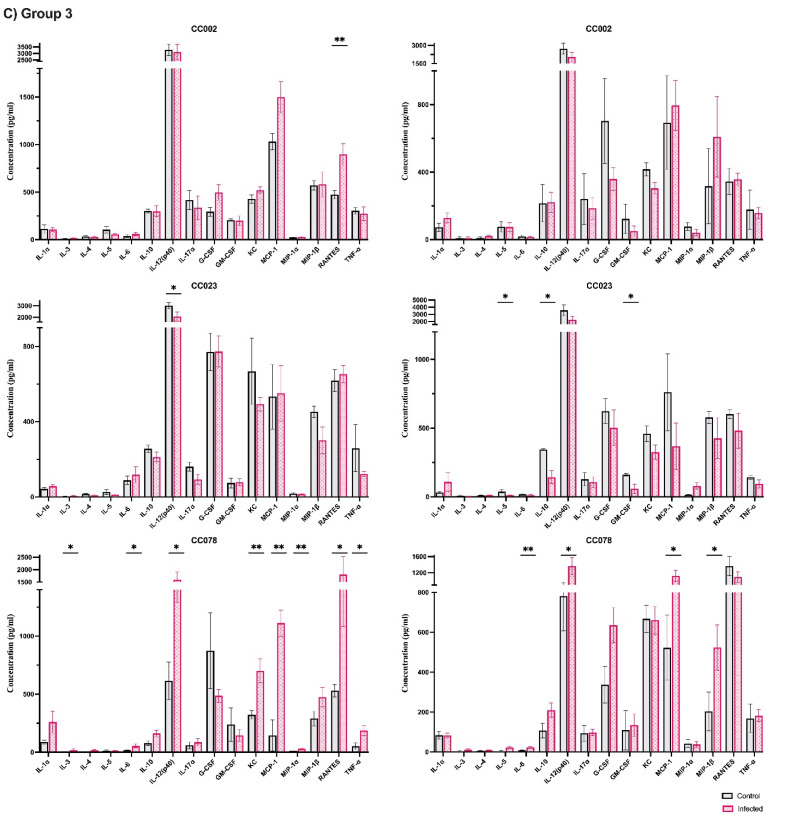
Summary snapshot of cytokines and chemokines significantly produced in response to TMEV at two time points post-injection. Strain-specific cytokines and chemokines were produced at significantly different levels in infected compared to PBS-injected mice (Mean ± S.E.M.; * *p* < 0.05, ** *p* < 0.01, *** *p* < 0.001). We classified the strains according to their phenotypic response to TMEV, as described in the legend of Figure 3: Group 1 (**A**), Group 2 (**B**), and Group 3 (**C**). The 23 cytokines and chemokines measured at 4 and 14 d.p.i. are available in Supplementary Figure S2A–D (23 cyto/chemokine levels for pre- and post-injection time points).

Here, we summarized the cytokine and chemokine profiles of those produced at significant levels compared to strain-specific control mice at both time points among the six different strains (Figure 4). Early post-injection responses at 4 d.p.i. showed strain-specific production of primarily pro-inflammatory cytokines and chemokines, e.g., IL-1α, IL-3, IL-5, IL-6, IL-12(p40), G-CSF, KC, MCP-1, MIP-1α, RANTES, and TNF-α. For example, infected mice from strain CC078 at 4 d.p.i. produced elevated levels (compared to PBS-injected CC078) for 8 of the 11 aforementioned cytokines and chemokines. Infected mice from the other strains produced elevated levels of one or two cytokines and chemokines. At the end of the acute phase (14 d.p.i.), most (13 out of 23) cytokines and chemokines remained at basal levels compared to control mice. The exceptions were IL-4, IL-5, IL-6, IL-10, IL-12(p40), IL-17α, GM-CSF, MCP-1, MIP-1β, and RANTES. Of these ten cytokines and chemokines, infected mice of strain CC078 had higher levels of four at 14 d.p.i. compared to PBS-injected CC078. Compared to strain-matched control mice, infected mice of the other strains produced elevated levels of up to three cytokines and chemokines, except for low levels of IL-17α in strain CC057. Strain CC023 produced significantly low levels of IL-12(p40) at 4 d.p.i. and IL-5, IL-10, and GM-CSF at 14 d.p.i. when compared to PBS-injected mice.

### 3.5. Gender-Specific Differences Were Identified for Certain Cytokine and Chemokine Levels at 4 and 14 d.p.i.

We sought to identify gender-specific differences in cyto/chemokine levels at 4 and 14 d.p.i. for each strain (Table 4). There were more gender differences at 14 d.p.i. compared to 4 d.p.i. Also, the chemokine Eotaxin was disproportionately represented as 8 of the 12 differences identified overall for both genders at both time points, and all strains except CC078. These gender differences were also related to control vs. infected mice of the same strain (Supplementary Figure S3). For example, at 4 d.p.i., Eotaxin levels were significantly higher in TMEV-infected vs. PBS-injected CC023 females; however, levels were significantly higher in PBS-injected (vs. TMEV-infected) CC023 males. By 14 d.p.i., female CC023 mice no longer showed a substantial difference in Eotaxin levels in control vs. infected mice. However, male PBS-injected CC023 continued to show significantly higher Eotaxin levels at 14 d.p.i. Furthermore, G-CSF levels at 4 d.p.i. were significantly lower in TMEV-infected males compared to PBS-injected or any females of strain CC027. At 14 d.p.i., this difference was even more pronounced.

### 3.6. Pre-Injection Serum Levels Serve as Predictive Markers for TMEV-Induced Phenotypes

We hypothesized that levels of cytokines and chemokines in serum correlate with TMEV-induced phenotypes during the acute phase of infection. We performed a stepwise regression analysis to identify significant relationships between pre-injection cytokine/chemokine levels and specific TMEV-induced phenotypes (Table 5). For all strains, we determined that pre-injection levels of IL-1β (*p* < 0.01) and TNF-α (*p* < 0.001) were associated with observations of decreased righting reflex. However, TMEV infection status, as a variable for stepwise regression, did not significantly correlate with decreased righting reflex.

Next, serum levels of TNF-α (*p* < 0.001), IL-1β (*p* < 0.01), and MIP-1β (*p* < 0.05) were associated with limb weakness. Though we sought to identify relationships between immune responses and paralysis, not all mice displayed limb paralysis during the acute phase of infection. Therefore, we included in our analysis only those strains that lost limb mobility: CC002, CC023, and CC078. We found associations between limb paralysis and IL-9 (*p* < 0.05) among the three strains. TMEV infection (but not PBS-injection) was associated with both limb weakness (*p* < 0.01) and limb paralysis (*p* < 0.001) observed by 14 d.p.i. Finally, we did not identify associations between the seizure phenotype and immune responses during the acute phase since few mice evaluated had seizures.

**Figure 5:**
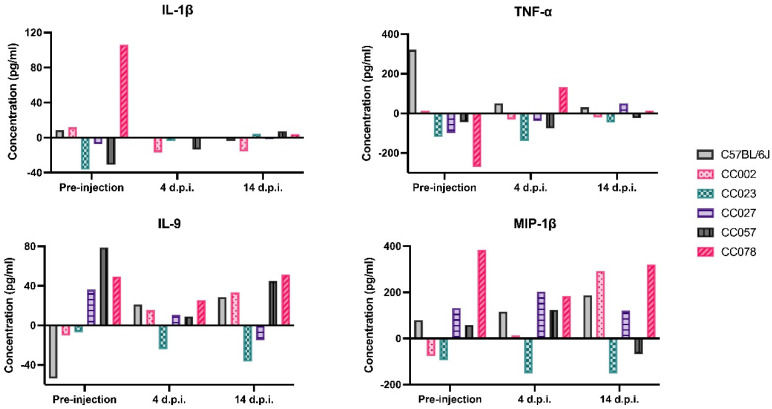
Strain-specific temporal changes in phenotype-associated cytokines and chemokines. To reflect average strain-specific differences produced throughout the acute phase, we subtracted average control levels from average infected levels respective to the observed time point. The y-axes demonstrate the differences among concentration levels for the selected cytokines and chemokines. Levels below the x-axis demonstrate that PBS-injected mice produced these cytokines at higher levels than TMEV-infected mice at that specific time point. Levels above the x-axis illustrate that TMEV-infected mice produced these responses at higher levels than PBS-injected mice. These cytokines and chemokines can be used as potential serum biomarkers for the different TMEV-induced phenotypes observed throughout the first 14 days of infection.

Serum levels for cytokines and chemokines varied across all time points (pre-injection, 4 d.p.i. and 14 d.p.i.) throughout the acute phase of infection (Figure 5; also, Supplementary Figure S2A–D). In general, these cytokines and chemokines were not found at uniformly high levels throughout the acute phase nor had a correlative relationship with the induced phenotype; rather, these levels fluctuated throughout the acute phase depending on the strain. In some cases, even prior to injection, mice from control and infected groups of the same strain did not have similar cytokine and chemokine levels due to the heterogeneity established by the CC strains. Because mice were randomly assigned to treatment groups, this observation reflected pre-existing individual-level variation; similar findings have been reported for plasma cytokine levels in humans, e.g., [52,53]. This variability, prior to injection with TMEV, could be hypothesized to be due to prior subclinical infections, different stress responses to transport stress (from their birth colony to our procedure room), behavioral aggression from new cage mates, puberty, etc. More relevant to acute-phase phenotypes was how these levels changed following the i.c. injection procedure, and thereafter.

The stepwise regression analyses identified four serum cyto/chemokines of interest as potential biomarkers for certain TMEV-induced phenotypes of acute disease. These included TNF-α, IL-1β, MIP-1β, and IL-9. Cytokines TNF-α and IL-1β, and the chemokine MIP-1β (induced by IL-1β), are generally considered pro-inflammatory; however, IL-1β and MIP-1β can also moderate inflammation. IL-9 is regarded as a pleiotropic cytokine with either pathogenic or beneficial effects, depending on the broader context. Accordingly, molecular networks regulated by (or involving) these cyto/chemokines influence differences in neurological sequelae and, by extension, the differences distinguishing Groups 1, 2, and 3.

## 4. Discussion

In this study, we identified the effects of the intracranial injection procedure on strain-specific immune responses. We characterized temporal changes from baseline (pre-injection) to 14 d.p.i. levels of cytokines and chemokines in serum after TMEV infection. Additionally, we found associations between pre-injection immune responses in serum and specific neurological sequelae induced by acute TMEV infection. Thus, we identified serum biomarkers for viral-induced neurological disease.

TMEV has been critical for modeling human neurological damage associated with viral infections, such as demyelinating disease and epilepsy. Intracranial inoculation of TMEV infects resident CNS cells, such as oligodendrocytes, astrocytes, microglia, and macrophages, which prompt immune responses to restrict viral replication [29,30,54]. The source of cytokine and chemokines in serum stem from the CNS or peripheral lymphoid organs as a result of viremia, which occurs following i.c. injection and activates the immune system. The immune response may cause severe bystander damage during acute infection by eliciting a rapid and prolonged inflammatory response resulting in neurological symptoms. Both TMEV-susceptible (e.g., SJL/J) and TMEV-resistant (e.g., C57BL/6J) inbred mouse strains mount a strong pro-inflammatory cytokine response within the CNS during the acute phase of infection, for example as shown by increased transcript levels for cytokines IFN-γ, IL-1, IL-6, IL-12p40, and TNF-α. By the middle of the acute phase (around 8 d.p.i.), SJL/J mice continue to exhibit a highly pro-inflammatory immune response throughout the CNS, unlike B6 mice. Interestingly, also at day 8, SJL/J mice develop high levels of TGF-b following TMEV infection, which is thought to inhibit cytotoxic T cells [55]. Pro-inflammatory inducers, including MCP-1, MIP-1α, and RANTES, have been found upregulated in the cerebrospinal fluid (CSF) of TMEV-infected (compared to PBS-injected) SJL/J mice [56]; pro-inflammatory cytokines IL-1β, IL-6, and TNF-α have also been observed to be elevated in serum from TMEV-infected BALB/c [57], CBA [44], and SJL/J [25] mice. In fact, the pro-inflammatory cytokines IL-6 and IL-1β have critical roles in the pathogenic immune responses leading to TMEV-induced demyelinating disease in SJL/J mice [26,58]. Differences in cyto/chemokine expression continue throughout TMEV infection, ultimately contributing to dramatically different disease outcomes.

Before determining which immune responses were attributable solely to TMEV, we accounted for the effects of the i.c. injection TMEV (Table 2) as prior TMEV studies have largely overlooked differences in immune profiles in response to i.c. injection. Furthermore, the fact that these differences were reflected in serum levels almost immediately after injection underscores the traumatic nature of the injection procedure. The widespread strain-specific immune responses to this procedure, based on our findings, are of importance when evaluating subsequent responses to TMEV infection as it is possible the injection procedure itself could set the stage for immune-mediated damages to the CNS.

We characterized baseline responses using pre-injection levels of immune response to identify specific cytokine and chemokine fluctuations during this critical period of symptom susceptibility. Our analysis indicated that in infected mice, a mix of IL-3, IL-5, IL-6, IL-10, IL-13, IL-12(p40), IL-17α, G-CSF, GM-CSF, KC, MIP-1α/β, and RANTES levels differed throughout 4 d.p.i. and 14 d.p.i. (Table 3). These responses are distinct from other TMEV studies as they do not consist of typical IL-1, IL-6, or TNF-α. There are cytokines and chemokines within the same strain that overlap in both PBS- and TMEV-infected mice. These can be considered inherent to the strain itself, as we have normalized the levels produced according to each strain’s baseline (pre-injection) response.

We further depicted serum profiles at specific post-injection time points (4 d.p.i. and 14 d.p.i.), including those residual responses to the i.c. injection. We revealed distinctive cytokine and chemokine patterns produced in response to TMEV for each strain, independent of similar phenotypic response profiles (e.g., Groups 1, 2, and 3; Figure 3 and Figure 4) based on prior findings from virus-infected CC strains [25]. We focused on profiling serum cyto/chemokine responses in greater detail to identify the potential utility of CC strains as models for specific diseases.

We determined that at 4 d.p.i., CC027 mice (Group 1) produced high levels of cytokines and chemokines (IL-5, IL-12[p40] and RANTES) known for the cellular maintenance of eosinophils [59], chemoattraction of macrophages, and stimulation of dendritic cells [60] and other inflammatory cells [61], potentially contributing to viral encephalitis [62,63]. Another abundant chemokine in infected CC027 mice, RANTES, has previously shown neuroprotective effects in mice infected with West Nile Virus (WNV) by regulating the trafficking of leukocytes to the brain [64]. At 14 d.p.i., higher levels of IL-4 and IL-5 in infected mice suggested an attenuation of inflammatory responses, with improved cell survival and maturation of B cells and eosinophils [65] and increased growth of Th2-type cells [66]. IL-4 and IL-5 have been associated with positive outcomes to infection by Hepatitis B virus [67] and influenza [68] and aid in the clinical recovery of experimental autoimmune encephalomyelitis (EAE) models [69] of multiple sclerosis. Significant differences in G-CSF levels in control and infected females and males, for both time points, could indicate differences in the degree of overall inflammation (e.g., [70]). Gender differences in levels of G-CSF are not widely reported but could have far-reaching implications; therefore, this finding is worth further investigation. Overall, the acute phase immune profile for CC027 (Group 1) mice indicated resilience to viral-induced neurological dysfunction.

Mice from Group 2, strains B6 and CC057, developed seizures and mild encephalitis during the acute phase. B6 mice produced a response characteristic of a typical inflammatory milieu (IL-1α and IL-6 at 4 d.p.i. and RANTES at 14 d.p.i.), consistent with previous studies of TMEV-induced seizures [23]. CC057 mice had increased production of G-CSF, an inducer for regulated neutrophil trafficking from bone marrow with potentially protective effects against viral infections [71]. These mice also produced low pro-inflammatory cytokine IL-17α [72]. IL-17α can be protective by inducing secretion of G-CSF, or pathogenic if dysregulated, promoting the accumulation of neutrophils at the injection site [73,74]. In the case of CC057 mice, high levels of G-CSF prevailed, contributing to a protective effect that was likely key to the minimal clinical symptoms observed during the acute phase. Furthermore, CC057 PBS-injected males had significantly higher levels of IL-12 (p40) and Eotaxin at 14 d.p.i. compared to their infected counterparts. Sera levels of IL-12(p40) have been shown to differ by gender in humans with schizophrenia [75], as well as in mouse models of Alzheimer’s disease [76] and blood-brain barrier disruption [77]. B6 infected females, on the other hand, had significantly higher levels of Eotaxin at 14 d.p.i. Serum levels of Eotaxin in humans infected with WNV indicated immune responses differed by gender [78], suggesting a role in the physical manifestation and severity of the disease; gender differences in serum Eotaxin levels have also been observed in humans concerning allergic inflammation [79]. Additionally, mouse strain has been shown to influence gender differences in Eotaxin levels (e.g., [77]). Given the role of Eotaxin in immune diseases, and the fact that not all strains showed a gender bias in Eotaxin levels, this chemokine may play a subtle but important role in the variable appearance of TMEV-induced diseases.

Group 3 strains CC002, CC023, and CC078 developed limb paralysis after inducing unique strain-specific immune responses in serum. In the case of CC002 mice, 22 of the 23 cytokines and chemokines examined were maintained at basal levels at 4 d.p.i. and 14 d.p.i. The sole exception was RANTES, a chemokine attractor possessing the potential of being both neuroprotective or pathogenic [80]. At 4 d.p.i. we found high levels of RANTES in the serum of CC002 mice, implying very high levels were produced in the CNS. CC002 mice lost limb mobility early during the acute phase, suggesting axonal loss or death of motor neurons [23,81,82] and a likely pathogenic role for RANTES in this strain, supporting previous associations found with chronic TMEV-induced paralysis [25]. On the other hand, infected CC023 mice produced low levels of IL-12(p40) at 4 d.p.i. and low levels of IL-5, IL-10, and GM-CSF at 14 d.p.i. compared to their PBS-injected (control) counterparts. Low levels of IL-12(p40) have been linked to less-severe clinical disease after infection by mouse hepatitis virus [63]. HIV-infected human cell lines showed no relationship between decreased IL-12 and production of the anti-inflammatory cytokine IL-10 [83]. However, low levels of IL-5 have been associated with disease progression in HIV infection [84,85], and while GM-CSF has been suggested as a possible treatment for neutropenia in AIDS patients, increased infiltration of inflammatory mediators in some patients is alarming [86]. Overall, the cyto/chemokine profile of TMEV-infected CC023 mice is reminiscent of HIV infection [87], characterized by insufficient protection from the virus and its physical consequences. Furthermore, the chemokine Eotaxin modulates susceptibility to disease progression following HIV infection [88,89]; levels of Eotaxin were significantly different between CC023 females and males, with control vs. infected mice having opposite levels. This was true at 4 d.p.i. for both genders, and for males only at 14 d.p.i.

Conversely, at 4 d.p.i., infected CC078 mice produced high levels of inflammatory inducers (IL-3, IL-12[p40], KC, MCP-1, MIP-1α, RANTES), and two of the three cytokines in the pro-inflammatory triad (IL-6 and TNF-α). IL-3 has been associated with relapse in EAE models of MS [90,91], and IL-12[p40] can induce tissue pathology and chronic inflammation [92,93]. Concurrent production of KC with MCP-1 may increase neutrophil recruitment involved in blood-brain-barrier (BBB) permeability, associated with increased morbidity and mortality [94]. Finally, MIP-1α/β and RANTES can bind to C-C chemokine receptor 5 (CCR5), rendering CCR5 unable to engage in defense against viruses [95,96,97]. Failure to clear the virus from infected cells can result in persistent production of IL-6 and TNF-α, which contribute to demyelinating diseases [97,98,99]. Indeed, levels of IL-6, IL-12(p40), and MCP-1 remained high at 14 d.p.i. in CC078 mice, along with high levels of MIP-1β. Furthermore, compared to males, infected females had significantly higher levels of RANTES at 4 d.p.i.; gender differences in RANTES expression have been described in humans as well, though typically, males have higher levels than females [100,101]. Overall, the immune responses observed in the CC078 strain were analogous to the cytokine storm observed in COVID-19 patients [102,103] and HIV patients [104,105].

Finally, we identified significant associations between pre-injection serum levels of cyto/chemokines and TMEV-induced phenotypes during the acute phase via stepwise regression modeling. This analysis enables the identification of a list of plausible explanatory variables associated with a given outcome. Here, the model revealed that TNF-α and IL-1β were predictive markers for delayed righting reflex responses. TNF-α and IL-1β were also associated with limb weakness, along with MIP-1β. The model also revealed a significant association between TMEV infection and limb weakness and paralysis. Furthermore, the model revealed IL-9 as a predictive marker for limb paralysis. In fact, IL-9 has been associated with autoimmunity in EAE models [106,107] and in the pathogenic induction of mast cells [108,109], which may influence BBB permeability and neurodegeneration in MS [110]. Levels of these predictive biomarkers varied across the strains in this study, underscoring how a single key contributor’s interaction can influence the outcome of a complex condition. By finding significant contributors to viral-induced phenotypes, which interact dynamically and fluidly, we may better understand why a single viral infection (or complex condition such as MS, ALS, and PD) manifests in multiple outcomes.

While levels present in the serum do not represent a perfect reflection of those in the CNS, we report serum cytokine levels rather than CNS levels to compare systemic immune differences without being limited to the immune-privileged CNS environment. Thus, these serum biomarkers reflect the overall immune environment in response to TMEV infection. From the perspective of animal model research, measuring and comparing cytokine and chemokine levels in serum rather than directly in CNS tissues is a more tenable solution for developing longitudinal immune profiles without requiring excessive animal numbers. Moreover, serum measurements provide a translational perspective, for instance, relevant to humans with conditions or infections affecting the CNS. Blood sampling is less invasive, with fewer side effects and risks than CSF sampling via lumbar puncture, which carries a higher chance of adverse events such as headaches and hemorrhages [111]. The work described in this study represents the first to characterize the longitudinal immune profiles in serum of genetically diverse mouse strains, valuable for less invasive model development involving neurological diseases.

## 5. Conclusions

In conclusion, we have, for the first time, identified the cytokines and chemokines produced in response to the intracranial injection process, allowing us to identify the immune responses attributed solely to TMEV. We have characterized longitudinal strain-specific systemic immune profiles underlying various TMEV-induced clinical symptoms. We identified significant associations between pre-injection serum levels and TMEV-induced phenotypes during the acute phase, thereby identifying cytokines and chemokines as predictive markers for acute viral-induced disease symptoms. Importantly, these biomarkers can be evaluated via serum, offering the possibility of a valuable and cost-effective approach for prognostic testing in humans. Further quantification and characterization of specific immune cells within serum and CNS will augment these findings and represent a critical need to explore central detrimental mechanisms involving sera and CNS pathological cytokine and chemokine induction. Overall, the findings reported here provide insight into the complex interactions of the immune response in the pathogenesis of viral-induced neurological disease, necessary to improve model fidelity and development of preventive treatments associated with human neurological disease.

## Figures and Tables

**Table 1:** Mice were allocated to treatment groups after weaning at three to four weeks of age.

Complete Mouse List						
Strain	Gender	Cohort A		Cohort B		Total
		PBS	TMEV	PBS	TMEV	
C57BL/6J	F	3	4	4	4	15
	M	3	4	4	4	15
CC002	F	2	4	3	4	13
	M	3	4	3	4	14
CC023	F	4	4	3	4	15
	M	9	6	3	4	22
CC027	F	3	4	3	4	14
	M	3	4	3	4	14
CC057	F	4	4	3	4	15
	M	3	4	3	5	15
CC078	F	3	3	3	7	16
	M	3	4	3	4	14
Total		43	49	38	52	182

**Table 2:** Highlighted are cytokines and chemokines produced in response to the i.c. injection. We summarized the cytokine and chemokine responses induced by the injection procedure, identified via paired difference analysis between pre-injection and post-injection (4 and 14 d.p.i.) levels, as described in Figure 2A. The immune responses to the injection varied by mouse strain at each time point. Individual changes to each exposure group can be found in Supplementary Table S1.

Cytokines and Chemokines Affected by Intracranial Injection throughout Post-Injection Timepoints																								
Strain	D.P.I.	IL-1α	IL-1β	IL-2	IL-3	IL-4	IL-5	IL-6	IL-9	IL-10	IL-12(p40)	IL-12(p70)	IL-13	IL-17α	Eotaxin	G-CSF	GM-CSF	IFNɣ	KC	MCP-1	MIP-1α	MIP-1β	RANTES	TNF-α
C57BL/6J	4																							
	14																							
CC002	4																							
	14																							
CC023	4																							
	14																							
CC027	4																							
	14																							
CC057	4																							
	14																							
CC078	4																							
	14																							

**Table 3:** Acute phase immune responses varied by strain and infection status. We identified strain-specific differences in immune response between 4 and 14 d.p.i. that were attributable to TMEV infection or, in the case of those observed in PBS-injected mice, inherent to the strain from the injection procedure. Cytokines and chemokines induced by the stress of the injection procedure itself were eliminated from consideration (Table 2) by normalizing post-injection levels to the strain-specific pre-injection (baseline) levels.

Cytokine and Chemokine Changes throughout 4 d.p.i. and 14 d.p.i.		
Strain	Control	Infected
C57BL/6J	Eotaxin	IL-5 Eotaxin GM-CSF
CC002	IL-6 IL-12(p40) KC MIP-1β RANTES	IL-3 IL-6 IL-12(p40) IL-17α G-CSF RANTES
CC023	IL-12(p70)	IL-5 IL-6 IL-13 KC MIP-1α
CC027	IL-4 IL-17α GM-CSF IFN-γ	IL-3 IL-10 IL-17α G-CSF GM-CSF KC RANTES
CC057	IL-6 IL-12(p40)	IL-6 IL-12(p40) MIP-1β
CC078	GM-CSF KC MCP-1 MIP-1α RANTES	IL-12(p40)

**Table 4:** Certain cyto/chemokine levels in control vs. infected mice were significantly different for females and males of different strains. Cells are highlighted according to the *p*-value of significance.

Gender-Specific Differences among Cytokine and Chemokine Levels across the Acute Phase of Infection						
Strain	Female		Male		*p*-Value	
	4 d.p.i.	14 d.p.i.	4 d.p.i.	14 d.p.i.		
C57BL/6J		Eotaxin			**0.05**	*
CC002			Eotaxin ^s^		**0.01**	**
CC023	Eotaxin		Eotaxin ^s^	Eotaxin ^s^	**0.001**	***
CC027	IL-12(p40)	Eotaxin	Eotaxin	G-CSF ^s^	**0.0001**	****
			G-CSF ^s^			
CC057				IL-12(p40) ^s^ Eotaxin ^s^		
CC078	RANTES					

^s^ Those cyto/chemokines with higher levels in PBS-injected mice than in TMEV-infected mice.

**Table 5:** Analyses identified cytokines and chemokines of interest as potential biomarkers for certain TMEV-induced phenotypes of acute disease.

Stepwise Regression Analysis Output per Phenotype					
Phenotype	Variables	Estimate	Std. Error	*p*-Value	Significance
Reflex	TNF-α	−0.078	0.015	1.28 × 10^−6^	***
	IL-1β	0.035	0.01	0.0015	**
Limb Paralysis	IL-9	−0.060	0.027	0.0305	*
	TMEV infection	−0.0120	0.028	1.47 × 10^−4^	***
Limb Weakness	TNF-α	−0.057	0.014	7.31 × 10^−5^	***
	IL-1β	0.031	0.010	0.0021	**
	MIP-1β	−0.020	0.008	0.0114	*
	TMEV infection	−0.073	0.026	0.0053	**

* *p* < 0.05, ** *p* < 0.01, and *** *p* < 0.001.

## Data Availability

The data presented in this article are available in Supplementary Materials.

## Supplementary Materials

The following supporting information can be downloaded at: https://www.mdpi.com/article/10.3390/cells11132044/s1, Figure S1: 14 d.p.i. Frequency scores of neurological phenotypes; Figure S2 (A) Strain specific individual cytokine and chemokine concentration levels at Pre-injection for A cohort (mice studied until 4 d.p.i.). (B) Strain specific individual cytokine and chemokine concentration levels at 4 d.p.i. (C) Strain specific individual cytokine and chemokine concentration levels at Pre-injection for B cohort (mice studied until 14 d.p.i.) (D) Strain specific individual cytokine and chemokine concentration levels at 14 d.p.i.; Figure S3: 4 d.p.i. Cyto/chemokine sex differences between control and infected groups. * *p* < 0.05, ** *p* < 0.01, *** *p* < 0.001, **** *p* < 0.0001; Table S1: Strain specific paired difference analysis of cytokines and chemokines.
