# Supplementary material for: Serum Cytokines Predict Neurological Damage in Genetically Diverse Mouse Models
Source: Cells. 2022 Jun 28;11(13):2044. doi: 10.3390/cells11132044 (PMC9265636; doi:10.3390/cells11132044)
Supplement: Supplementary file 1 [file cells-11-02044-s001.zip › cells-1769544 proof sup .pdf]

Supplemental Statistical Methods

The data consisted of serum levels for twenty-three cytokines/chemokines in six different strains of mice, including TMEV-infected and PBS-injected from both sexes for each strain. Cytokine values were included for three different time points, i.e., pre-injection, 4d.p.i. and 14d.p.i. We also evaluated measurements of the phenotypic qualities like paralysis, weakness, righting reflex, etc. Cumulative scores for every phenotype reflected the combined scores for all neurological symptoms at 4d.p.i. and 14 d.p.i., coinciding with cytokine/chemokine level measurements. Progression scores reflected overall change in disease severity over the acute phase of infection (cumulative score at 14 d.p.i. minus cumulative score at 4 d.p.i.). Our primary objective was to identify any relationships with phenotype scores and changes in cytokine values over the three timepoints. In addition, we were also interested to know which cytokines/chemokines, alone or in combination with others, were most predictive of the progression score of each phenotype.

To evaluate how temporal immune responses were influenced by each host's genetic background, we measured and compared serum levels of cytokines and chemokines at three timepoints: pre-injection (baseline), 4d.p.i., and 14d.p.i. Performance of t test revealed statistically significant temporal production of cytokines and chemokines, including some produced in relation to residual responses from the intracranial injection and some in relation to TMEV. To address the goal of identifying the most important cytokines/chemokines influencing phenotypes, a stepwise regression analysis was performed using the progression scores of the phenotypes as the response and the PBS-injected status as well as the twenty-three cytokine levels as covariates.

For the statistical analyses, we used R software, version 4.0.3. As a data preprocessing step, we standardized the log transformed cytokine and chemokine measurements. For standardization, we deducted overall mean values from each of the cytokines and then we divided the differences by overall standard deviation. T-test was performed on the paired differences of chemokine measurements from pre-injection and 4 d.p.i. timepoints to understand the pattern of immune response changes between pre-injection and 4 d.p.i. timepoints. Similar steps were performed to compare the changes between pre-injected and 14 d.p.i. measurements. After the above-mentioned steps, we performed two sample t-test to compare the differences between 4 d.p.i. and 14 d.p.i. Two sample t test was then performed using both sets of paired differences to identify significant cytokines when comparing 4 d.p.i. vs 14 d.p.i. measurements. All the above-mentioned t tests were performed separately on control and infected mice. Significant cytokines both for control and infected mice were selected based on the 5% level of significance.

We adapted the "stepwise" function in R written by Paul A. Rubin from Michigan State University to perform the stepwise regression. An important parameter of the stepwise regression procedure is alpha, which acts as a stopping criterion that prevents further variables from being included or excluded. As done previously [1] (refer to pp. 320), we chose alpha to be 0.05 as the inclusion and exclusion criteria. The stepwise regression procedure involves a forward selection mechanism that starts with the intercept only model and proceeds according to the optimal stopping criterion to choose the final model. The stepwise regression model uses the progression score (14d.p.i. – 4d.p.i.) of each phenotype as response. Twenty-three cytokine levels measured at pre-injected stage and the PBS-injected status were used as covariates. The procedure identified relationships between pre-injection cytokine and chemokine measurements and phenotype progression scores. The regression model was also adjusted for PBS-injected status which was shown to be significantly associated with the progression scores in many strains.

[1] Li, J. Bile acid metabolites in early pregnancy and risk of gestational diabetes in Chinese women: A nested case-control study. *EBioMedicine*, **2018**, *35*, 317–324.

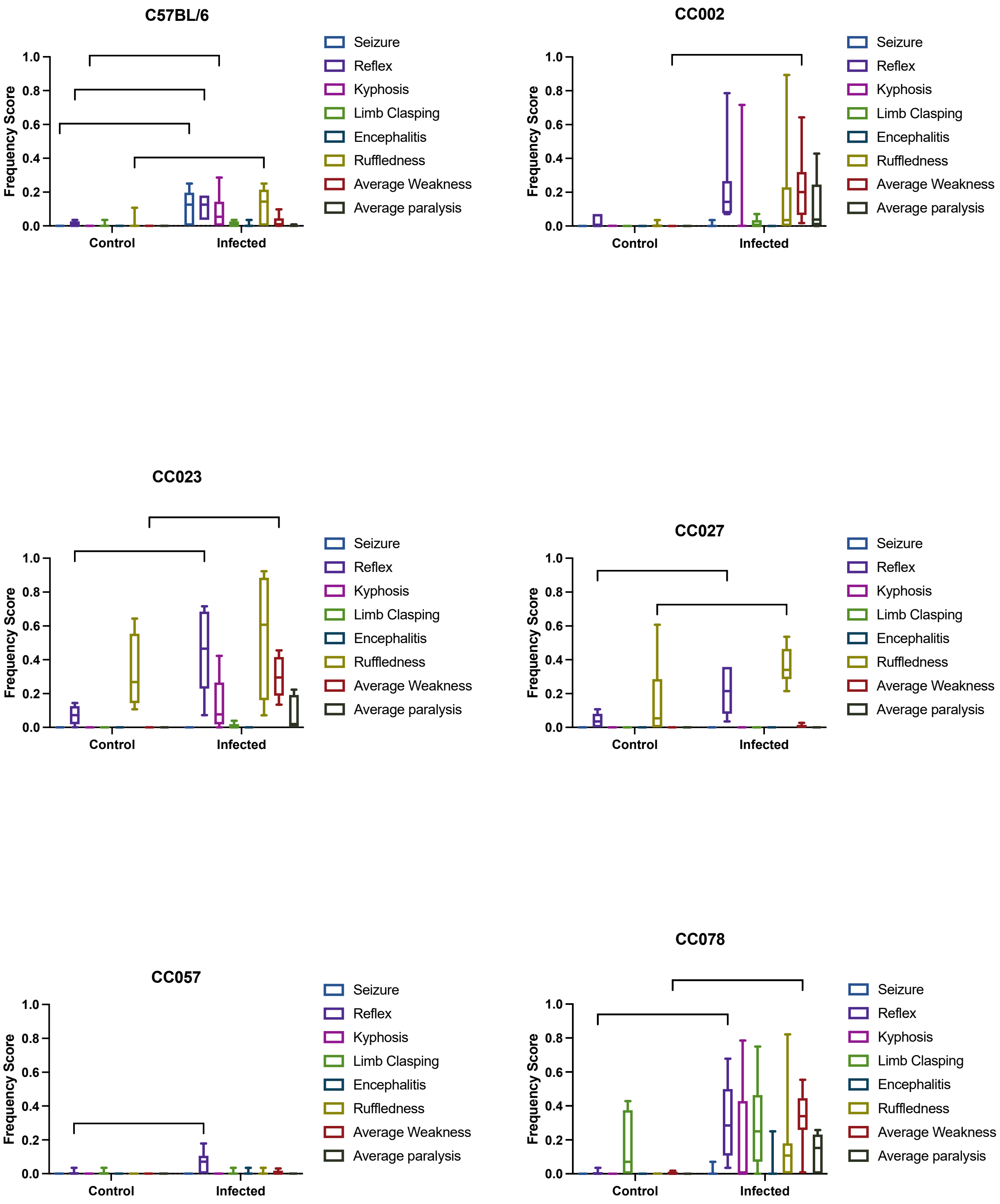

Figure S1. 14d.p.i. Frequency scores of neurological phenotypes.

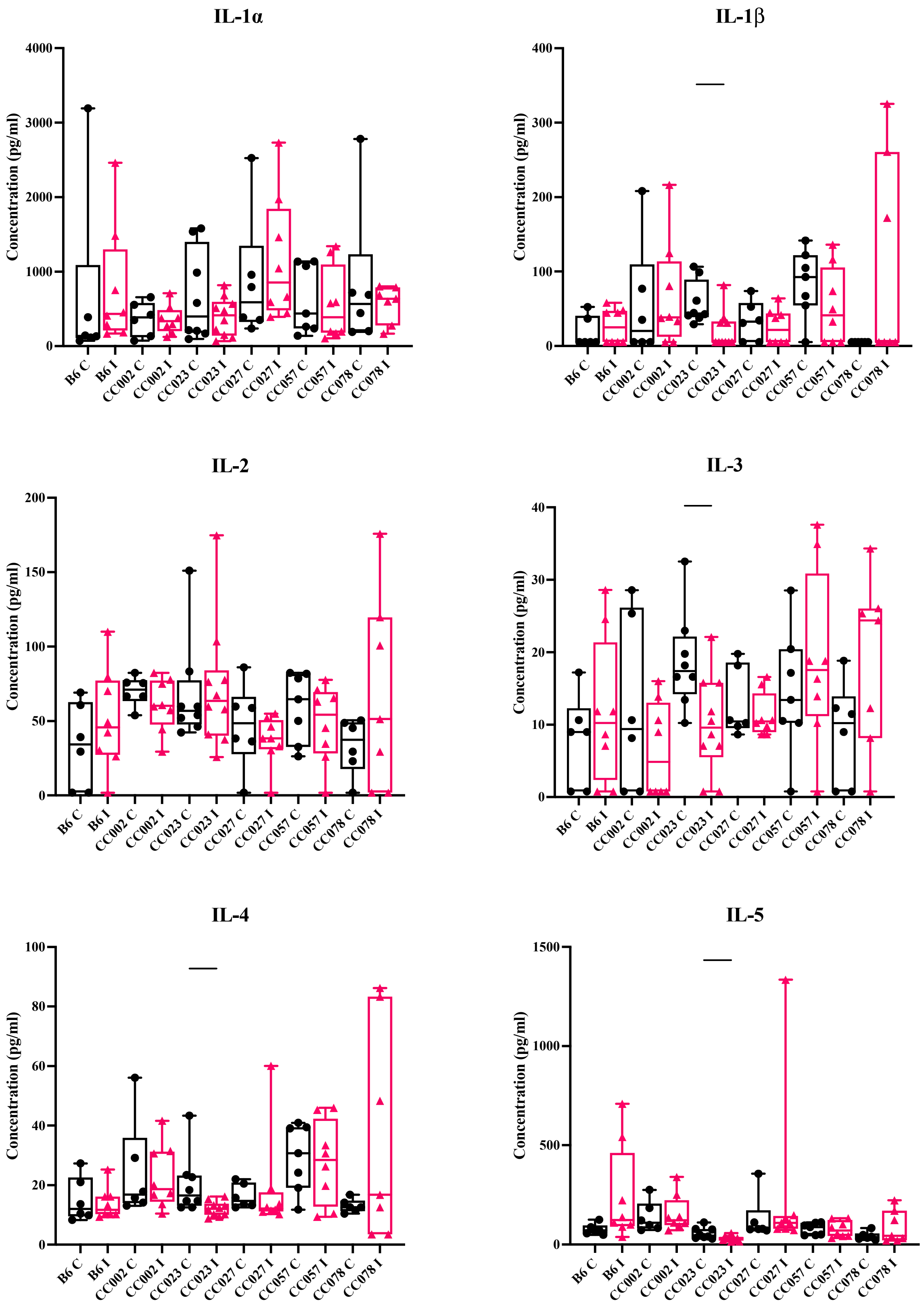
IL-1α
Concentration (pg/ml)
0
1000
2000
3000
4000
B6 C
B6 I
CC002 C
CC002 I
CC023 C
CC023 I
CC027 C
CC027 I
CC057 C
CC057 I
CC078 C
CC078 I
IL-1β
Concentration (pg/ml)
0
100
200
300
400
B6 C
B6 I
CC002 C
CC002 I
CC023 C
CC023 I
CC027 C
CC027 I
CC057 C
CC057 I
CC078 C
CC078 I
IL-2
Concentration (pg/ml)
0
50
100
150
200
B6 C
B6 I
CC002 C
CC002 I
CC023 C
CC023 I
CC027 C
CC027 I
CC057 C
CC057 I
CC078 C
CC078 I
IL-3
Concentration (pg/ml)
0
10
20
30
40
B6 C
B6 I
CC002 C
CC002 I
CC023 C
CC023 I
CC027 C
CC027 I
CC057 C
CC057 I
CC078 C
CC078 I
IL-4
Concentration (pg/ml)
0
20
40
60
80
100
B6 C
B6 I
CC002 C
CC002 I
CC023 C
CC023 I
CC027 C
CC027 I
CC057 C
CC057 I
CC078 C
CC078 I
IL-5
Concentration (pg/ml)
0
500
1000
1500
B6 C
B6 I
CC002 C
CC002 I
CC023 C
CC023 I
CC027 C
CC027 I
CC057 C
CC057 I
CC078 C
CC078 I

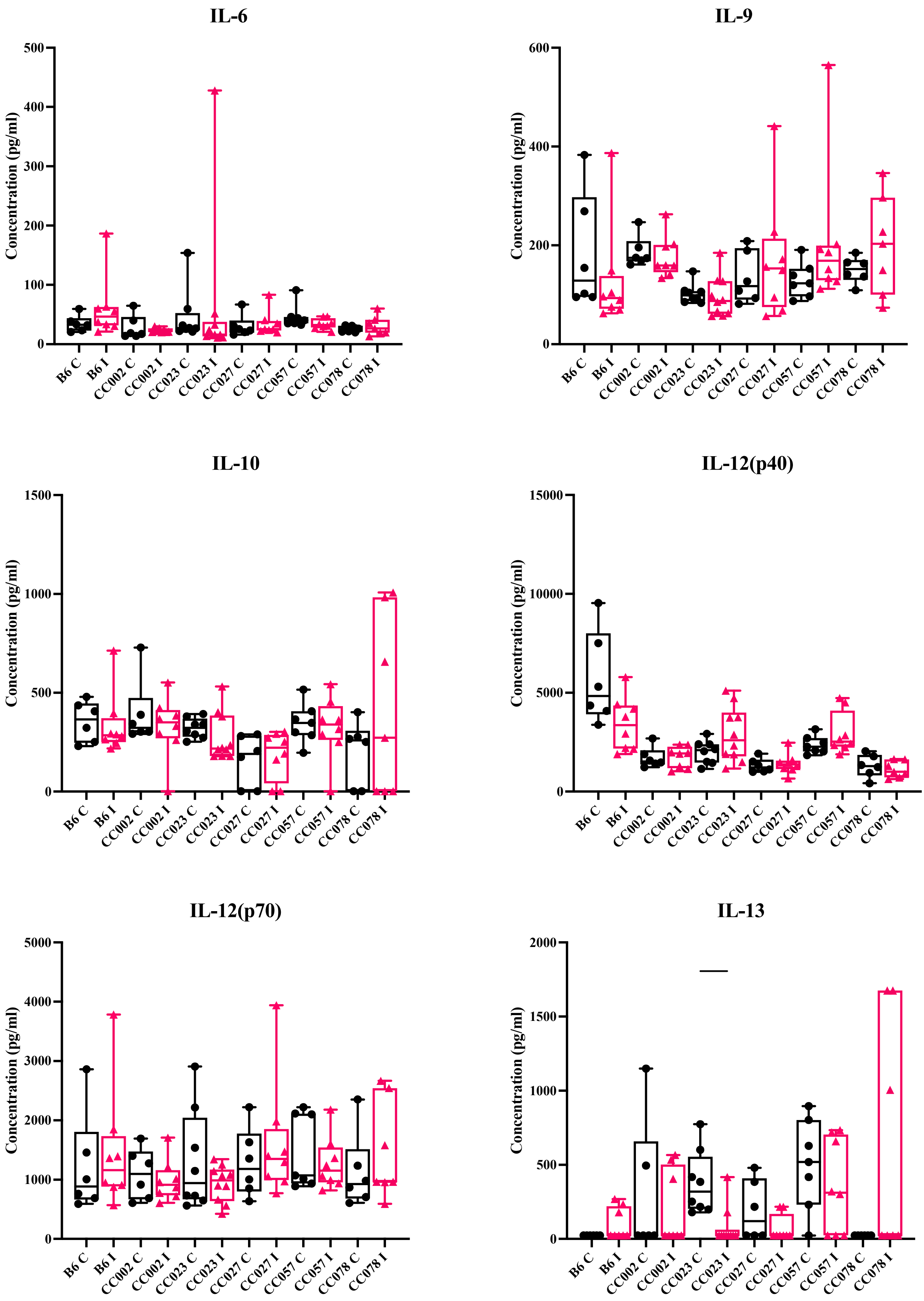
IL-6
IL-9
IL-10
IL-12(p40)
IL-12(p70)
IL-13
Concentration (pg/ml)
0
100
200
300
400
500
600
1000
1500
2000
3000
4000
5000
10000
15000
B6 C
B6 I
CC002 C
CC002 I
CC023 C
CC023 I
CC027 C
CC027 I
CC057 C
CC057 I
CC078 C
CC078 I

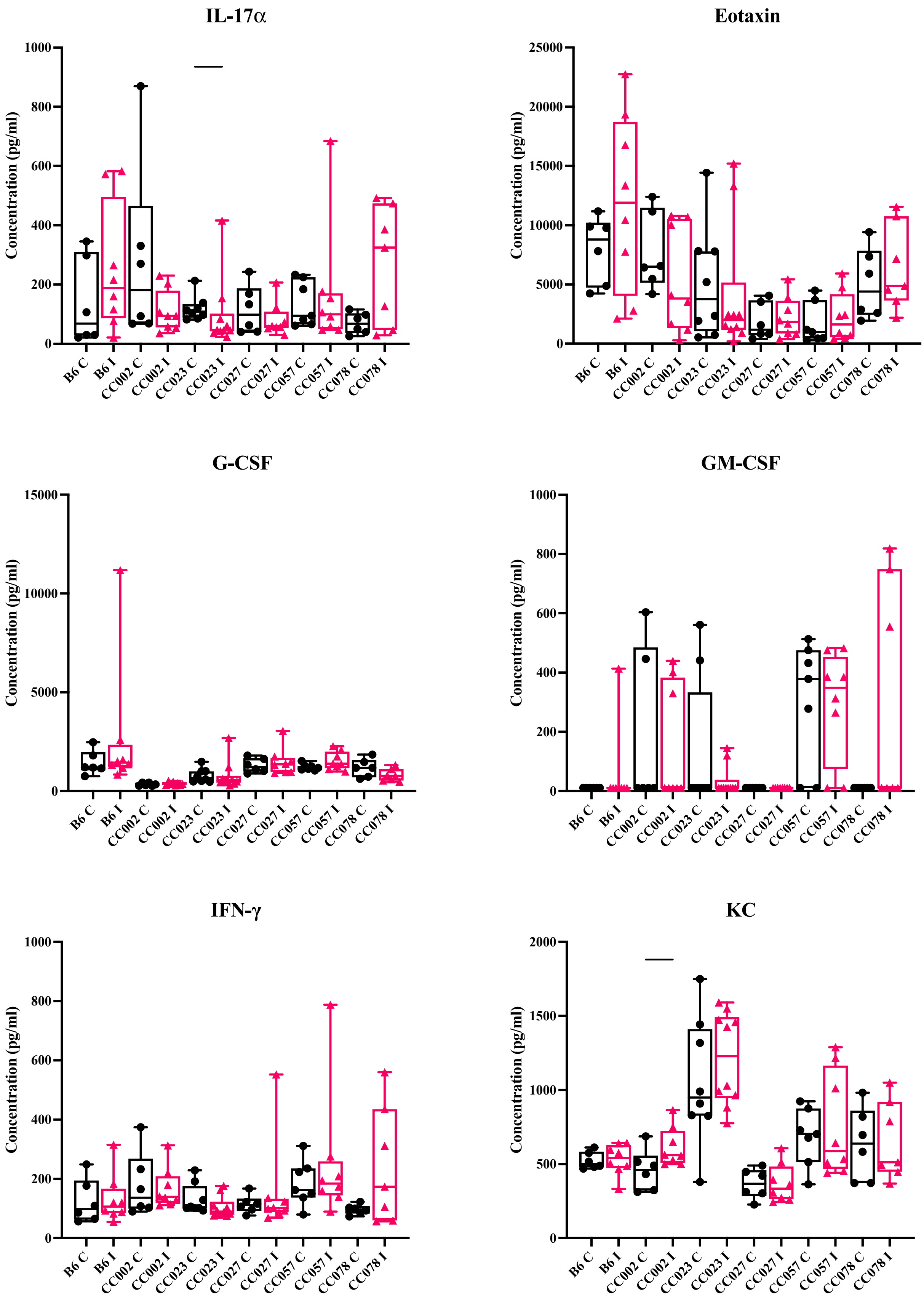
IL-17α
Concentration (pg/ml)
0
200
400
600
800
1000
B6 C
B6 I
CC002 C
CC002 I
CC023 C
CC023 I
CC027 C
CC027 I
CC057 C
CC057 I
CC078 C
CC078 I
Eotaxin
Concentration (pg/ml)
0
5000
10000
15000
20000
25000
B6 C
B6 I
CC002 C
CC002 I
CC023 C
CC023 I
CC027 C
CC027 I
CC057 C
CC057 I
CC078 C
CC078 I
G-CSF
Concentration (pg/ml)
0
5000
10000
15000
B6 C
B6 I
CC002 C
CC002 I
CC023 C
CC023 I
CC027 C
CC027 I
CC057 C
CC057 I
CC078 C
CC078 I
GM-CSF
Concentration (pg/ml)
0
200
400
600
800
1000
B6 C
B6 I
CC002 C
CC002 I
CC023 C
CC023 I
CC027 C
CC027 I
CC057 C
CC057 I
CC078 C
CC078 I
IFN-γ
Concentration (pg/ml)
0
200
400
600
800
1000
B6 C
B6 I
CC002 C
CC002 I
CC023 C
CC023 I
CC027 C
CC027 I
CC057 C
CC057 I
CC078 C
CC078 I
KC
Concentration (pg/ml)
0
500
1000
1500
2000
B6 C
B6 I
CC002 C
CC002 I
CC023 C
CC023 I
CC027 C
CC027 I
CC057 C
CC057 I
CC078 C
CC078 I

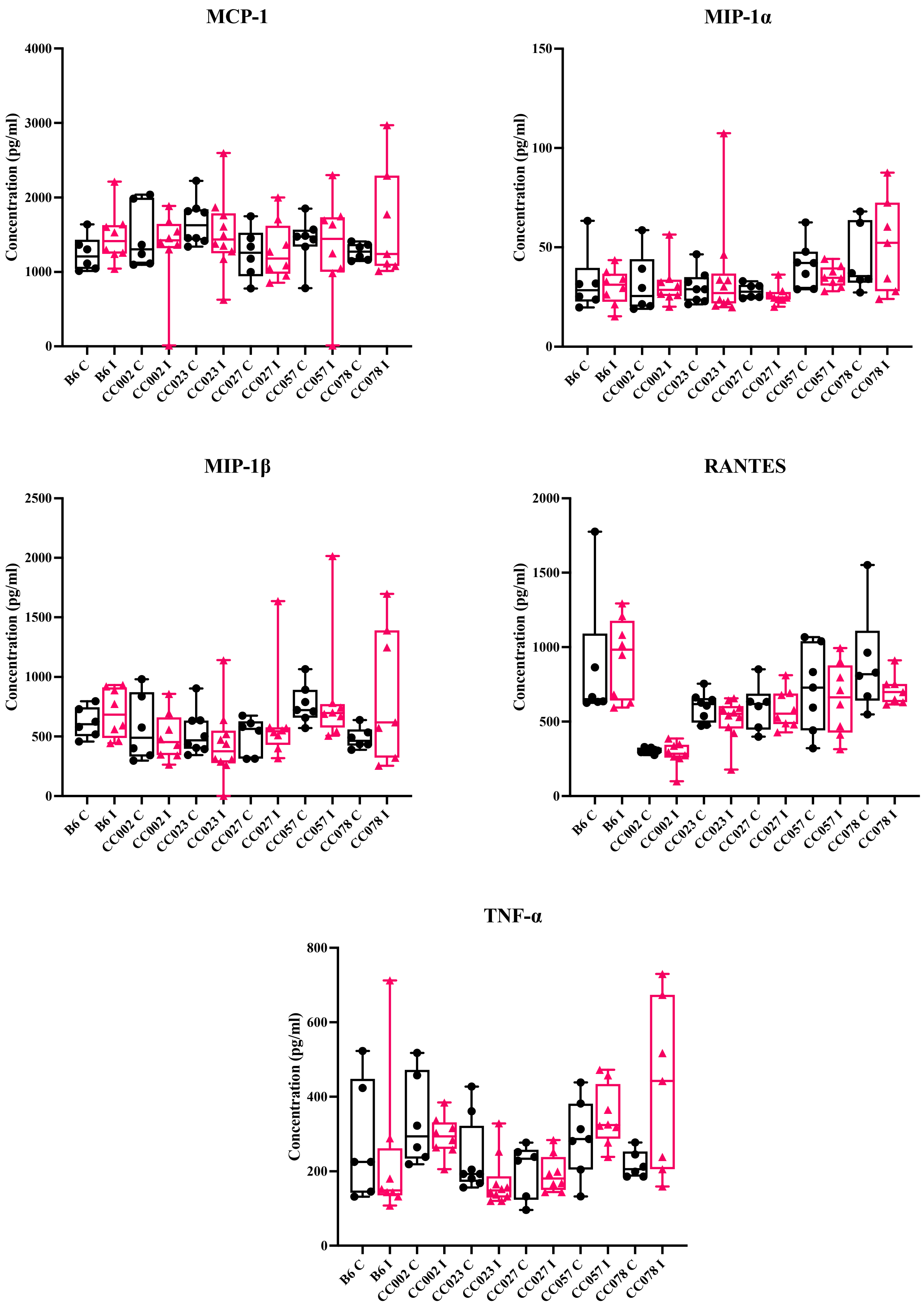

Figure S2A. Strain specific individual cytokine and chemokine concentration levels at Pre-injection for A cohort (mice studied until 4d.p.i.).

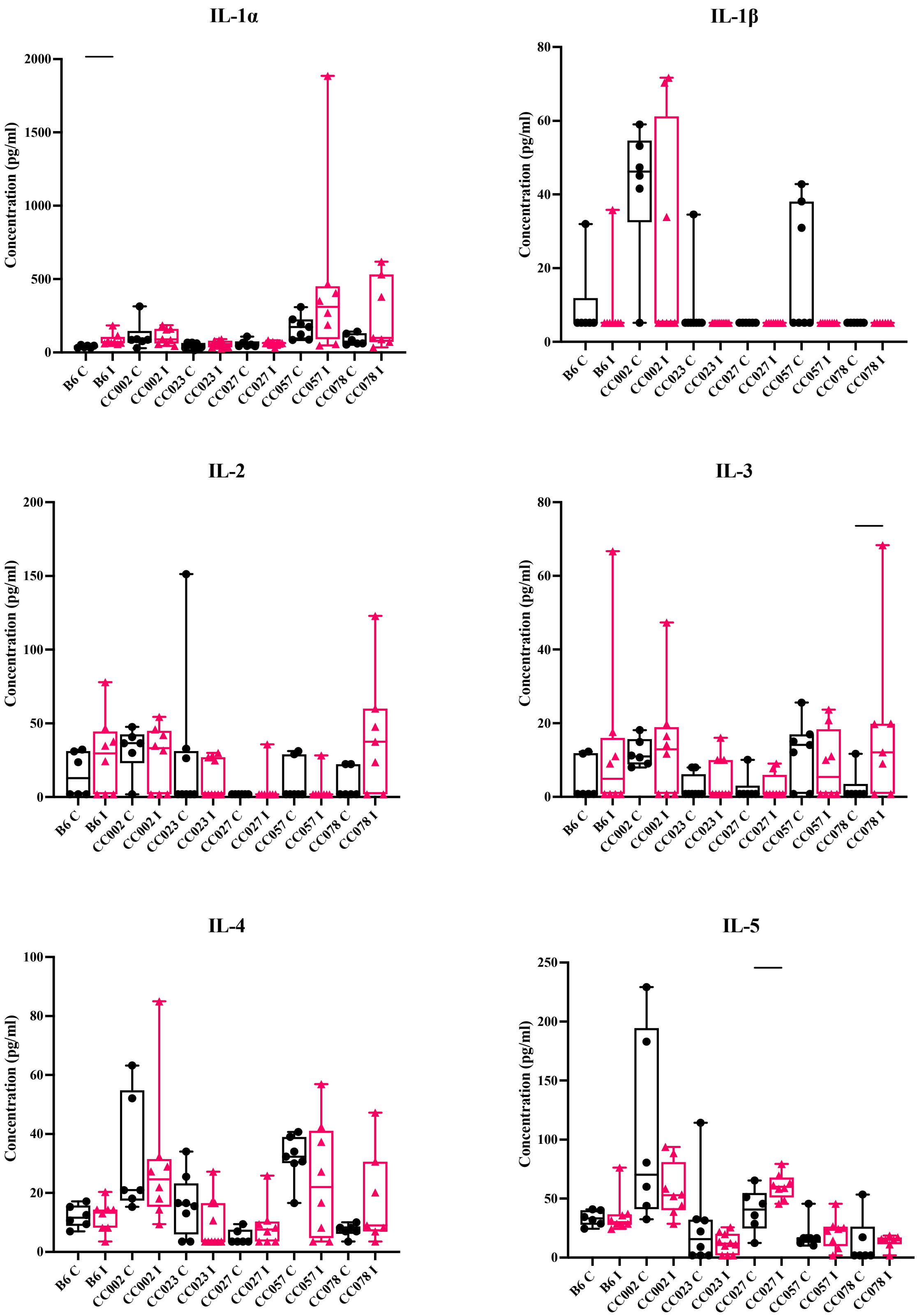
IL-1α
IL-1β
IL-2
IL-3
IL-4
IL-5
Concentration (pg/ml)
0
500
1000
1500
2000
20
40
60
80
50
100
150
200
250
B6 C
B6 I
CC002 C
CC002 I
CC023 C
CC023 I
CC027 C
CC027 I
CC057 C
CC057 I
CC078 C
CC078 I

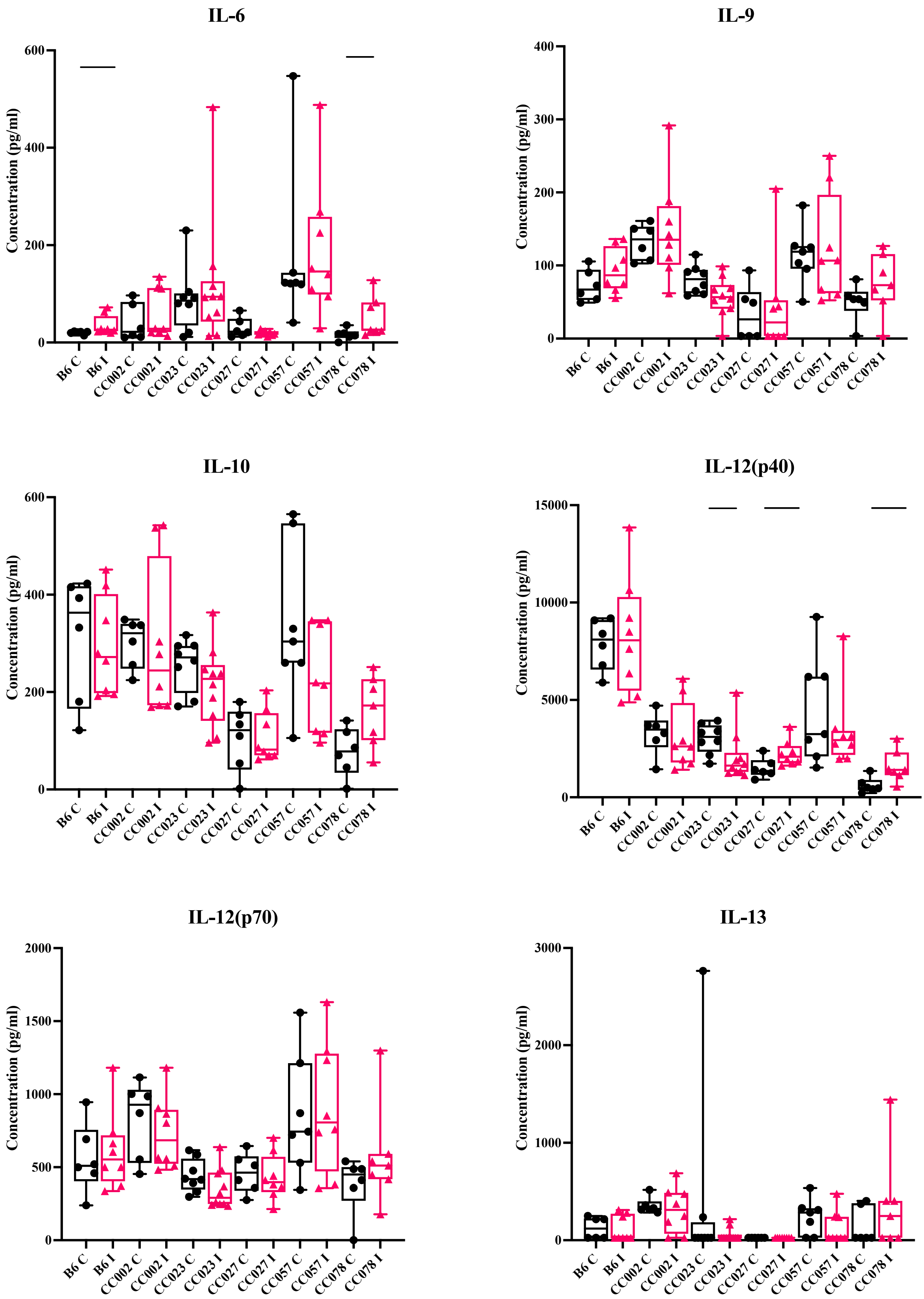
IL-6
IL-9
IL-10
IL-12(p40)
IL-12(p70)
IL-13
Concentration (pg/ml)
B6 C
B6 I
CC002 C
CC002 I
CC023 C
CC023 I
CC027 C
CC027 I
CC057 C
CC057 I
CC078 C
CC078 I

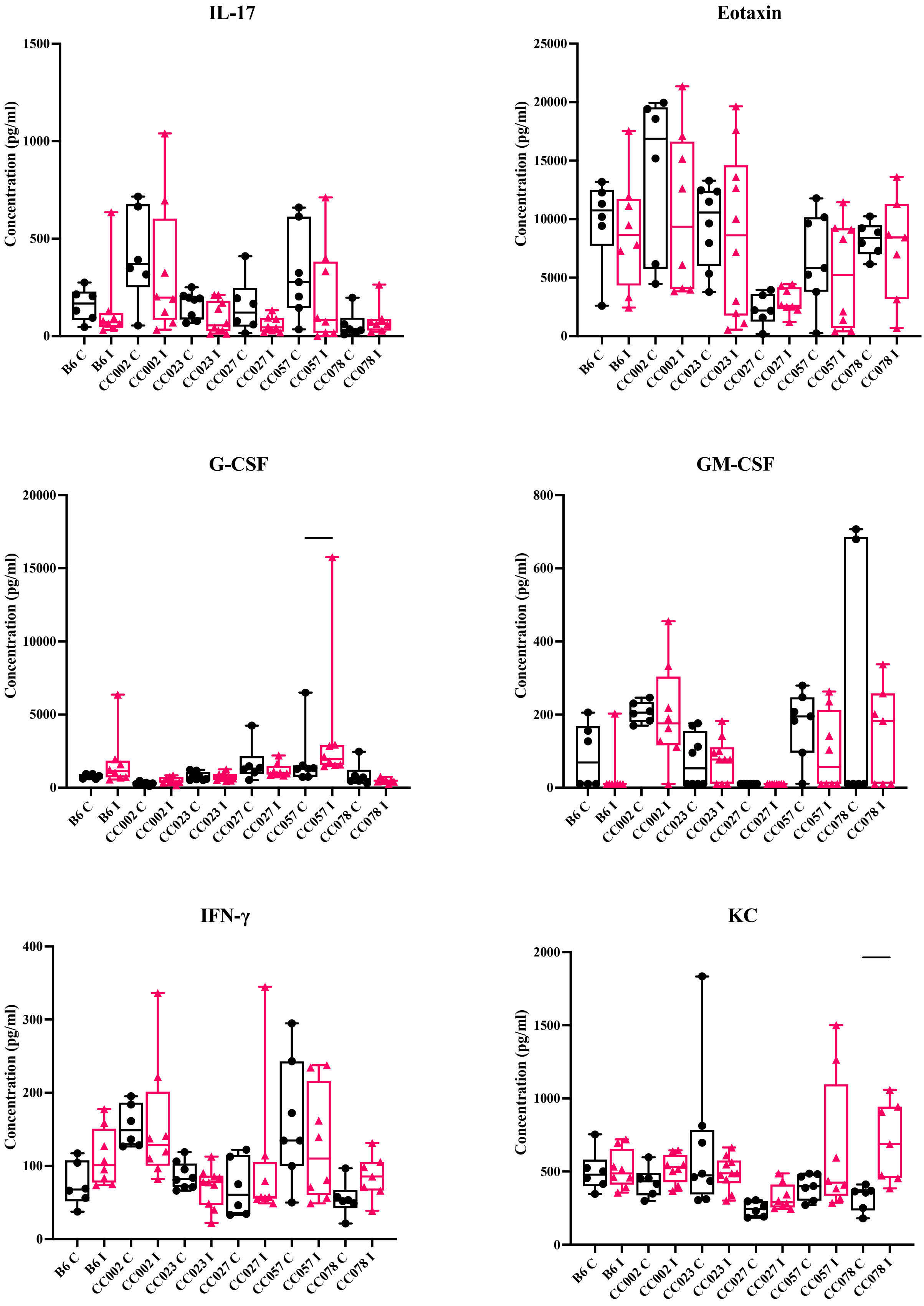
IL-17
Eotaxin
G-CSF
GM-CSF
IFN-γ
KC
Concentration (pg/ml)
0
500
1000
1500
5000
10000
15000
20000
25000
200
400
600
800
100
300
2000
B6 C
B6 I
CC002 C
CC002 I
CC023 C
CC023 I
CC027 C
CC027 I
CC057 C
CC057 I
CC078 C
CC078 I

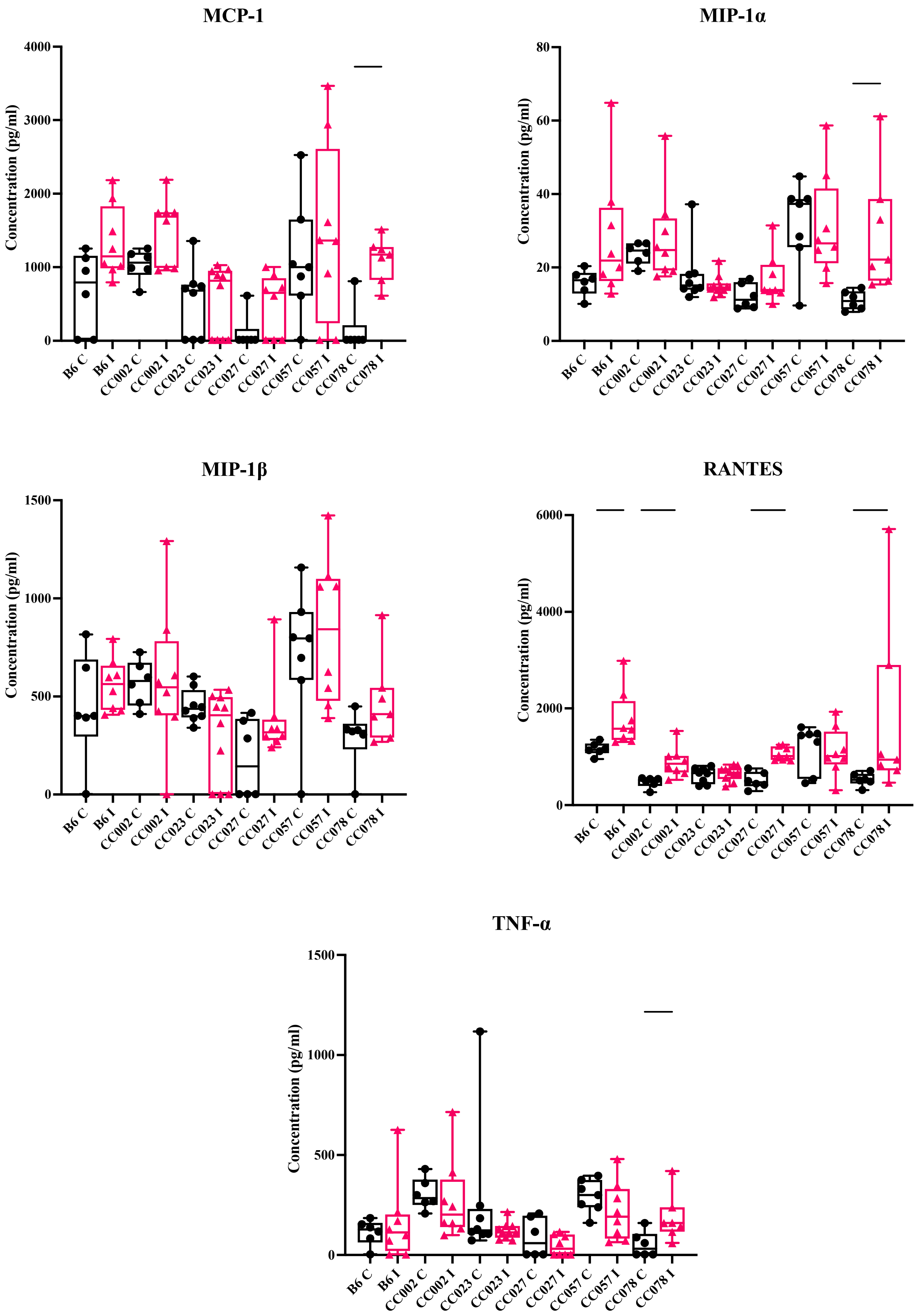

Figure S2B. Strain specific individual cytokine and chemokine concentration levels at 4d.p.i.

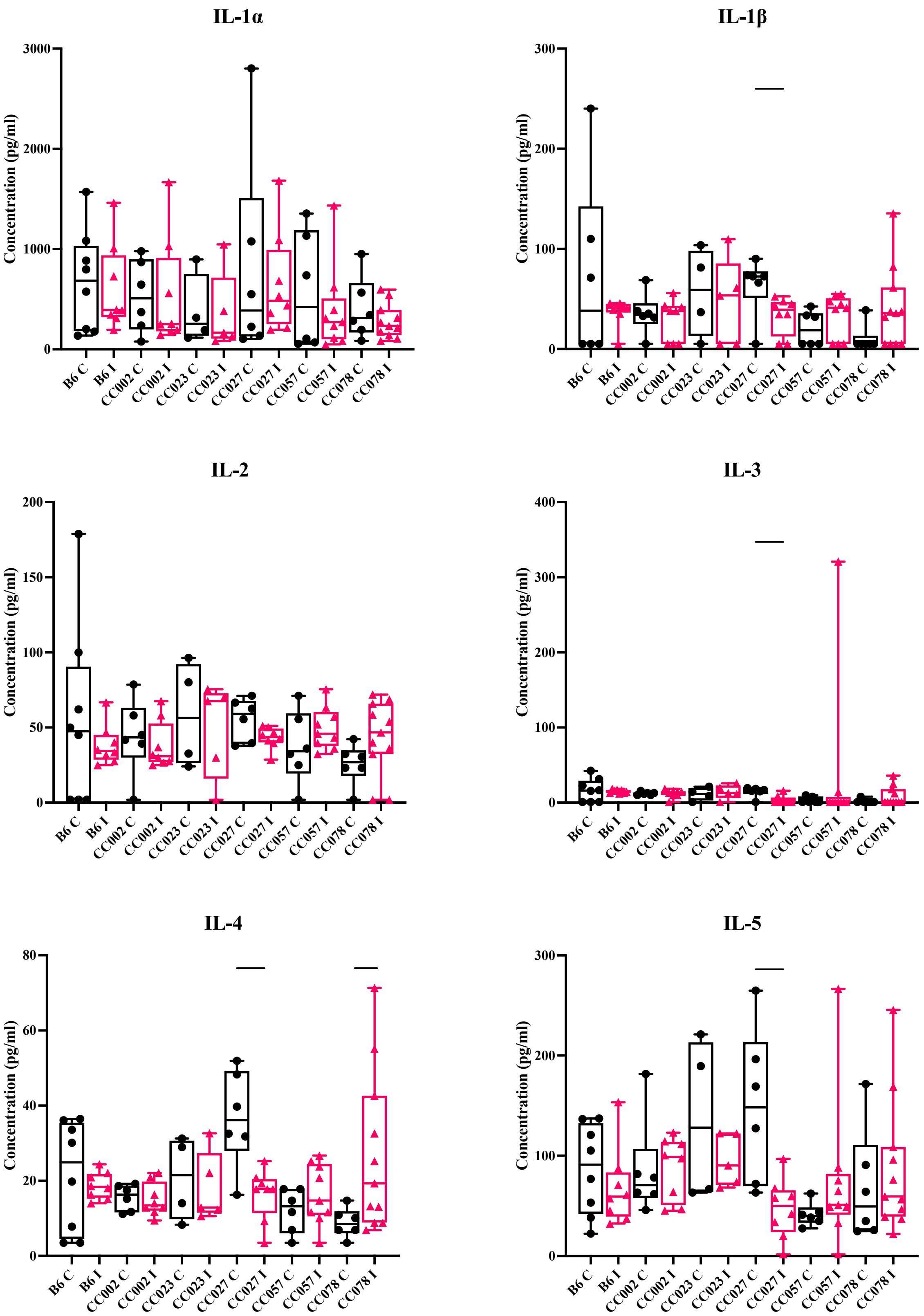

IL-1α
IL-1β
IL-2
IL-3
IL-4
IL-5
Concentration (pg/ml)
B6 C
B6 I
CC002 C
CC002 I
CC023 C
CC023 I
CC027 C
CC027 I
CC057 C
CC057 I
CC078 C
CC078 I

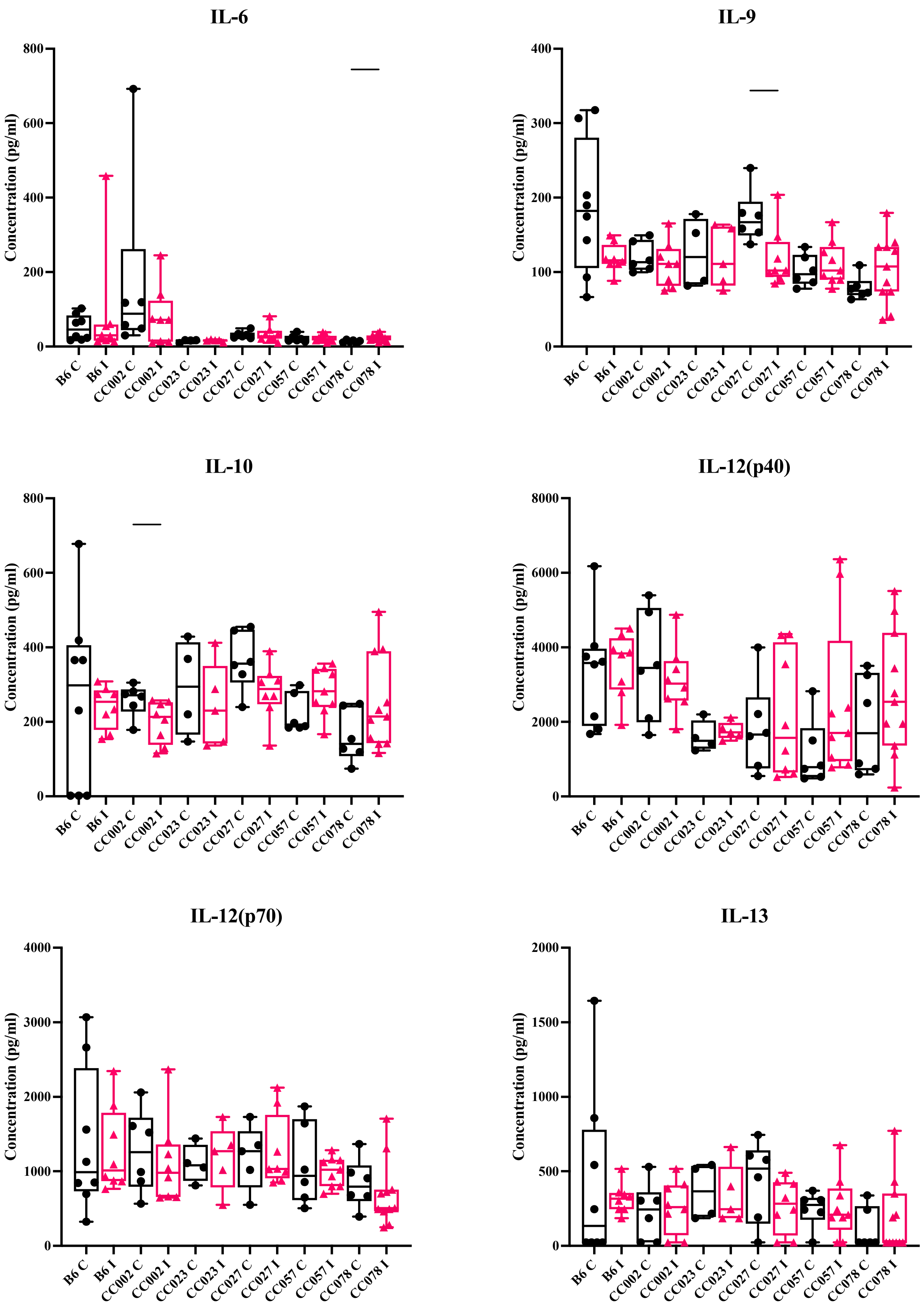
IL-6
IL-9
IL-10
IL-12(p40)
IL-12(p70)
IL-13
Concentration (pg/ml)
B6 C
B6 I
CC002 C
CC002 I
CC023 C
CC023 I
CC027 C
CC027 I
CC057 C
CC057 I
CC078 C
CC078 I

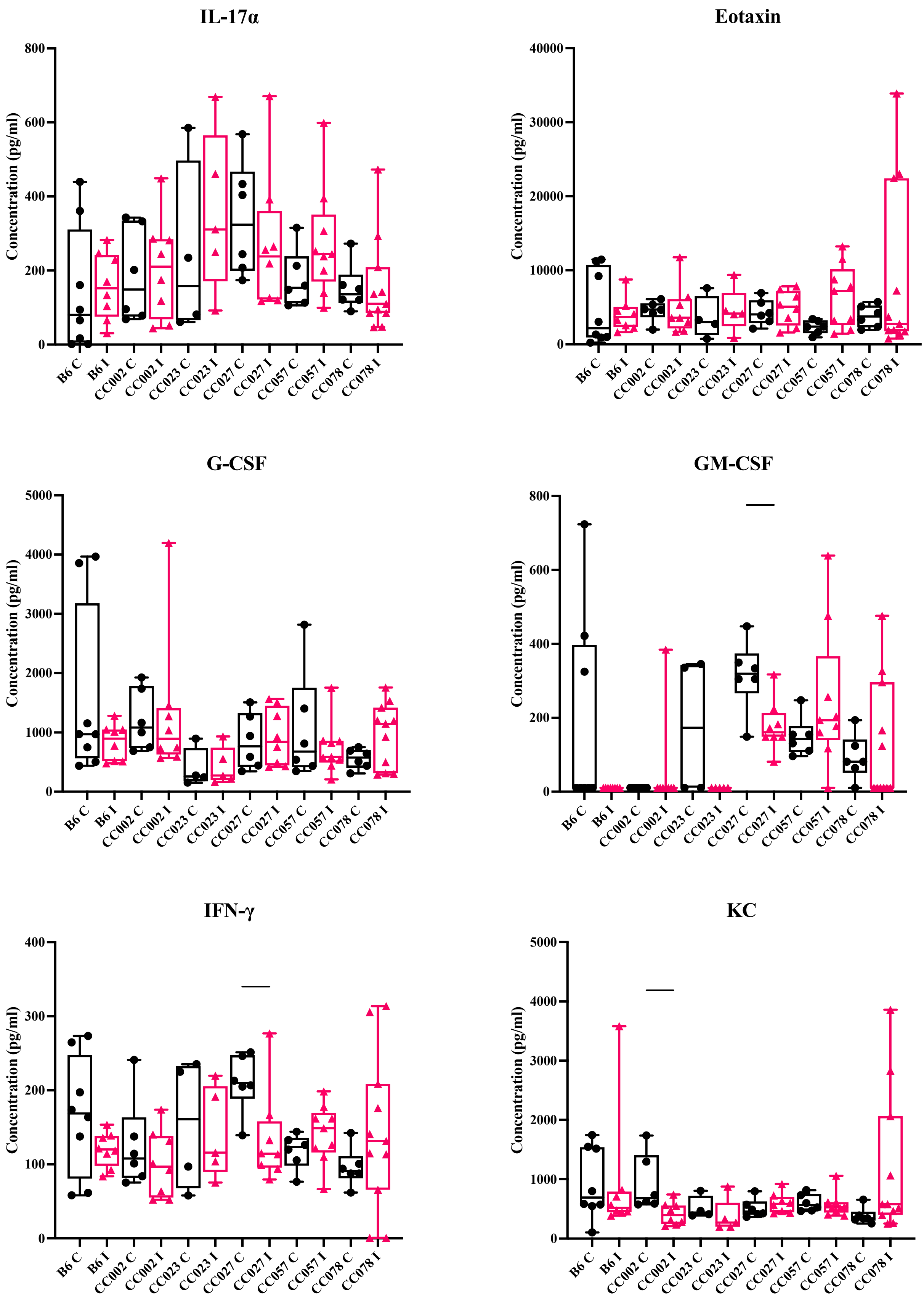
IL-17α
Eotaxin
G-CSF
GM-CSF
IFN-γ
KC
Concentration (pg/ml)
0
200
400
600
800
10000
20000
30000
40000
1000
2000
3000
4000
5000
100
300
B6 C
B6 I
CC002 C
CC002 I
CC023 C
CC023 I
CC027 C
CC027 I
CC057 C
CC057 I
CC078 C
CC078 I

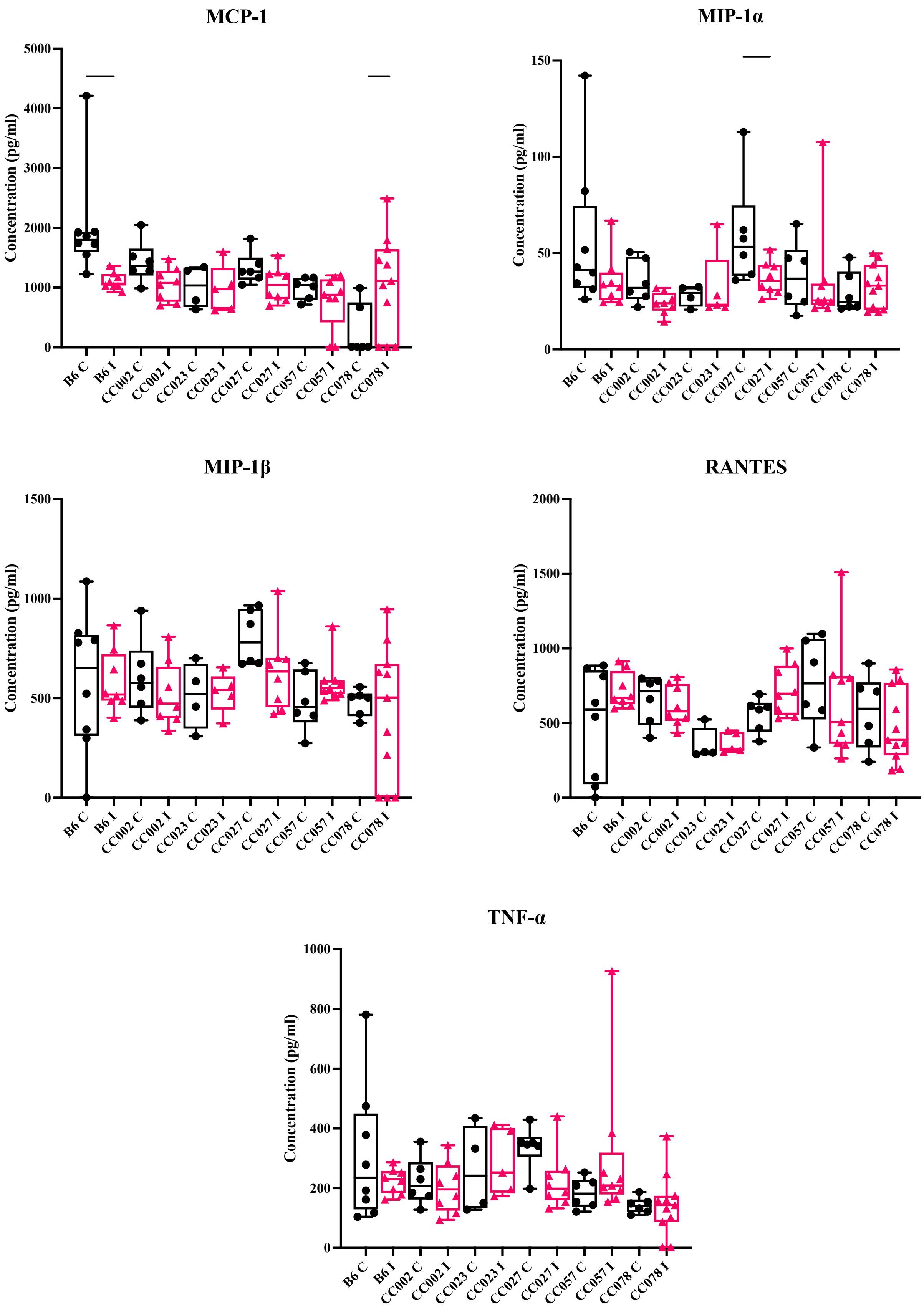

Figure S2C. Strain specific individual cytokine and chemokine concentration levels at Pre-injection for B cohort (mice studied until 14d.p.i.).

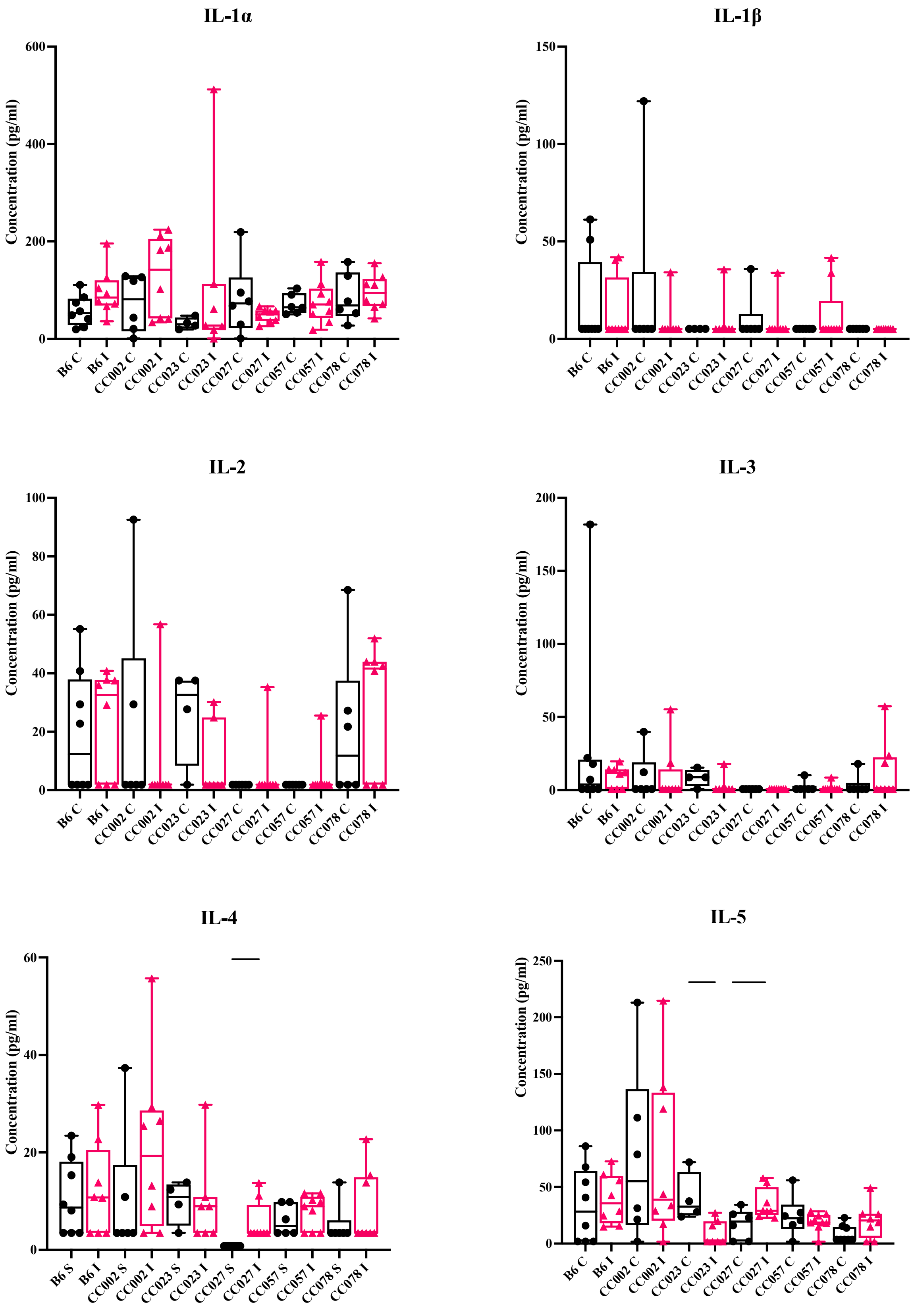
IL-1α
IL-1β
IL-2
IL-3
IL-4
IL-5
Concentration (pg/ml)
B6 C
B6 I
CC002 C
CC002 I
CC023 C
CC023 I
CC027 C
CC027 I
CC057 C
CC057 I
CC078 C
CC078 I
B6 S
CC002 S
CC023 S
CC027 S
CC057 S
CC078 S

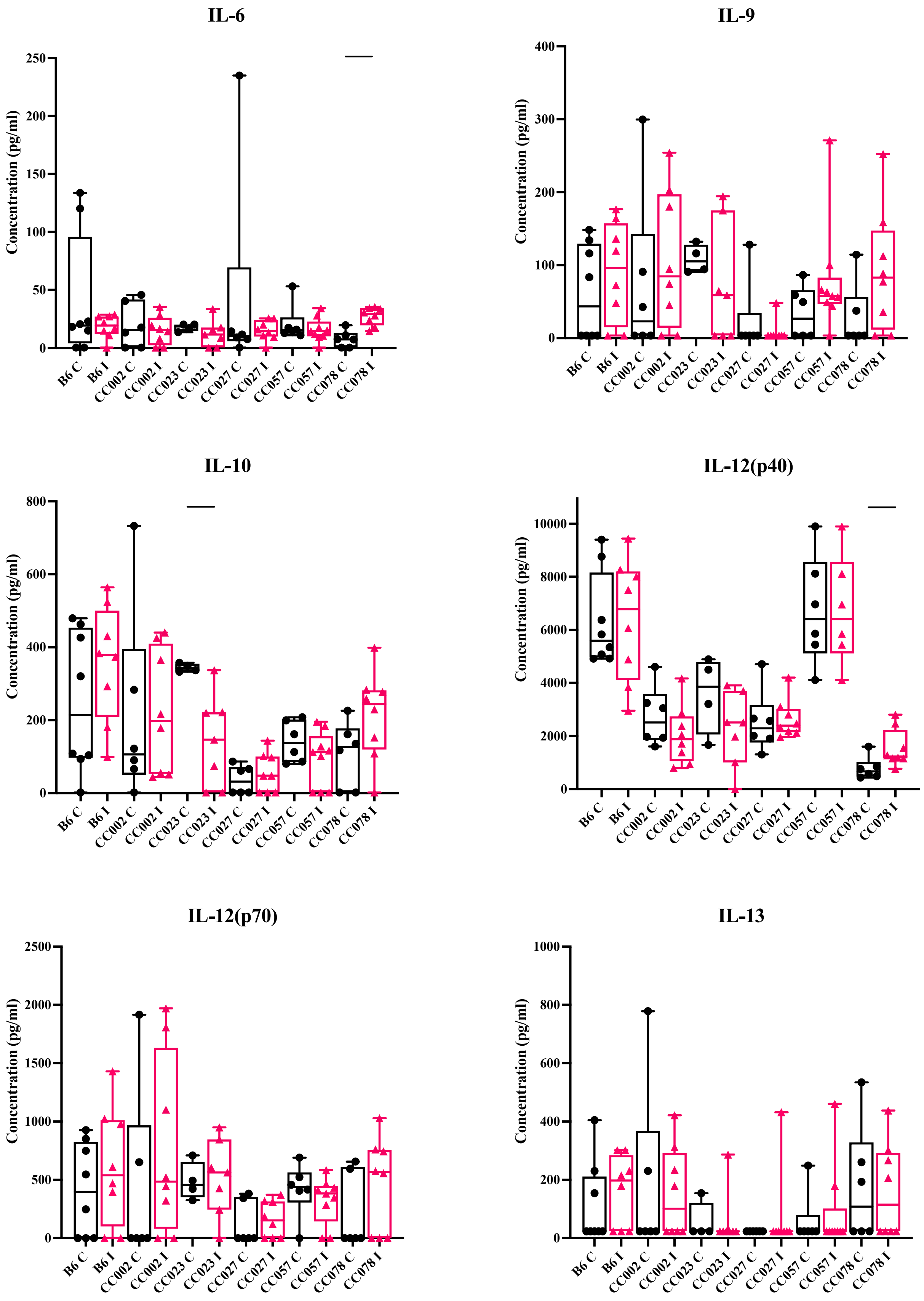
IL-6
Concentration (pg/ml)
0
50
100
150
200
250
B6 C
B6 I
CC002 C
CC002 I
CC023 C
CC023 I
CC027 C
CC027 I
CC057 C
CC057 I
CC078 C
CC078 I
IL-9
Concentration (pg/ml)
0
100
200
300
400
IL-10
Concentration (pg/ml)
0
200
400
600
800
IL-12(p40)
Concentration (pg/ml)
0
2000
4000
6000
8000
10000
IL-12(p70)
Concentration (pg/ml)
0
500
1000
1500
2000
2500
IL-13
Concentration (pg/ml)
0
200
400
600
800
1000

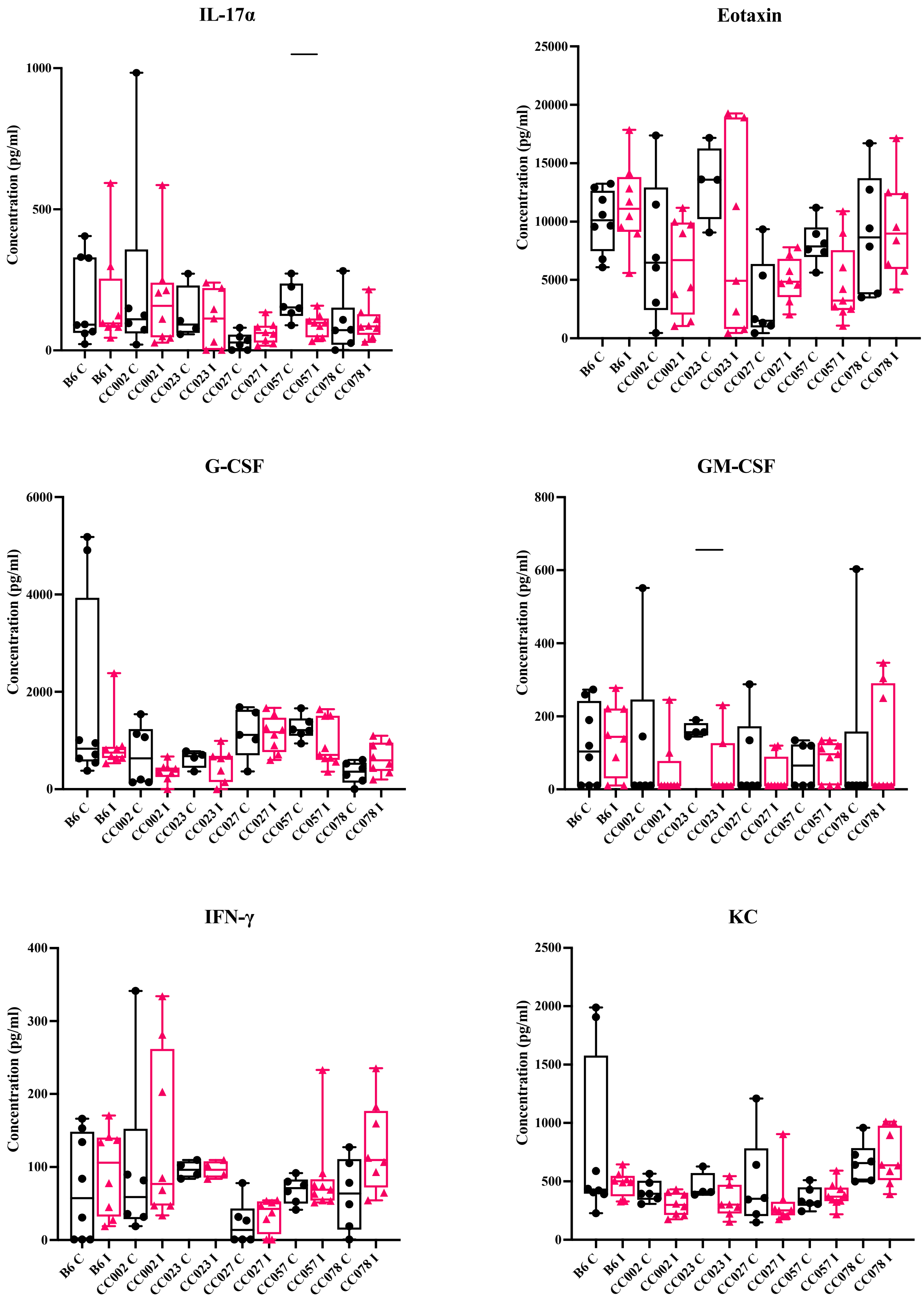
IL-17α
Eotaxin
G-CSF
GM-CSF
IFN-γ
KC
Concentration (pg/ml)
0
500
1000
5000
10000
15000
20000
25000
2000
4000
6000
200
400
600
800
100
300
1500
2000
2500
B6 C
B6 I
CC002 C
CC002 I
CC023 C
CC023 I
CC027 C
CC027 I
CC057 C
CC057 I
CC078 C
CC078 I

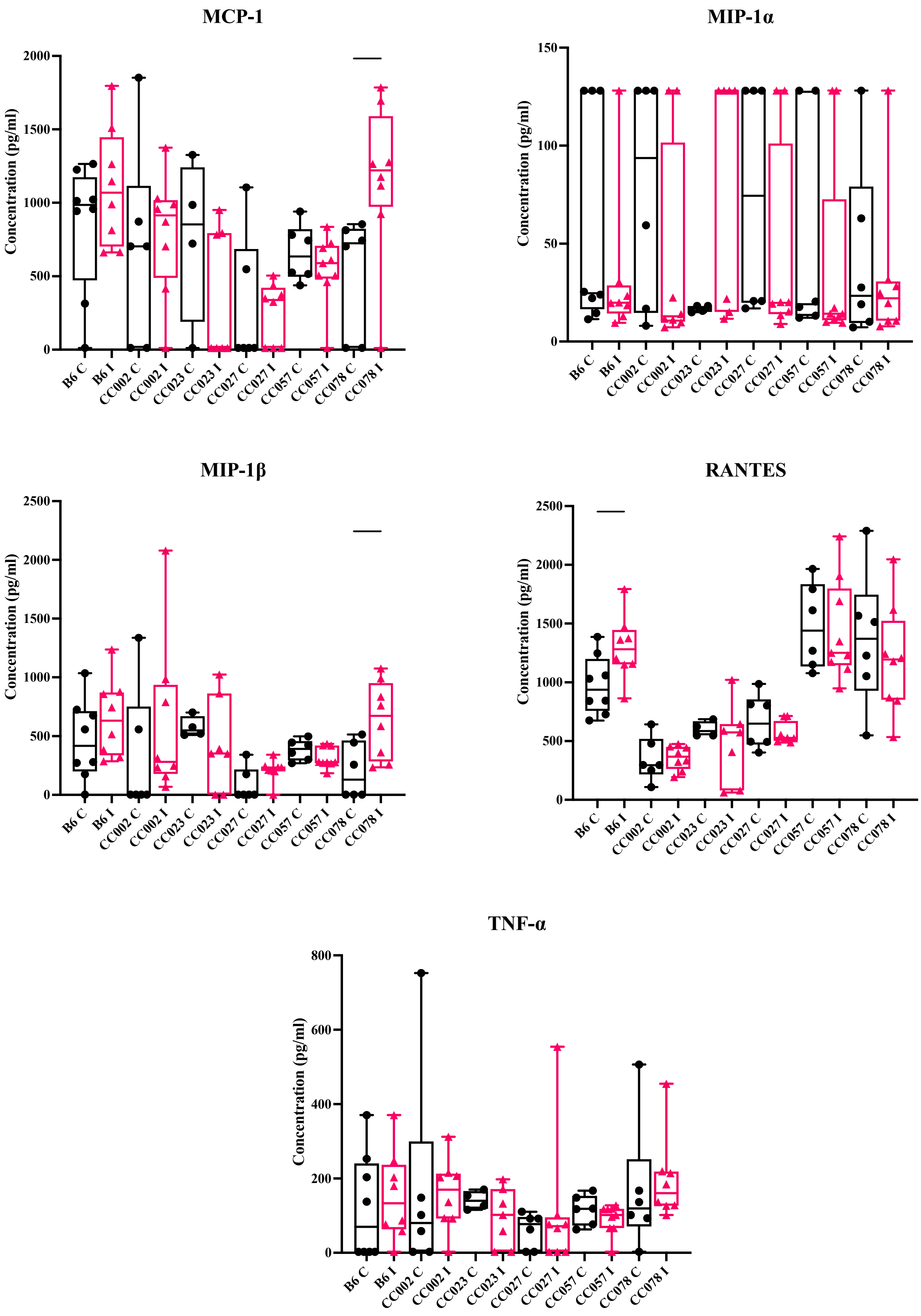

Figure S2D. Strain specific individual cytokine and chemokine concentration levels at 14d.p.i.

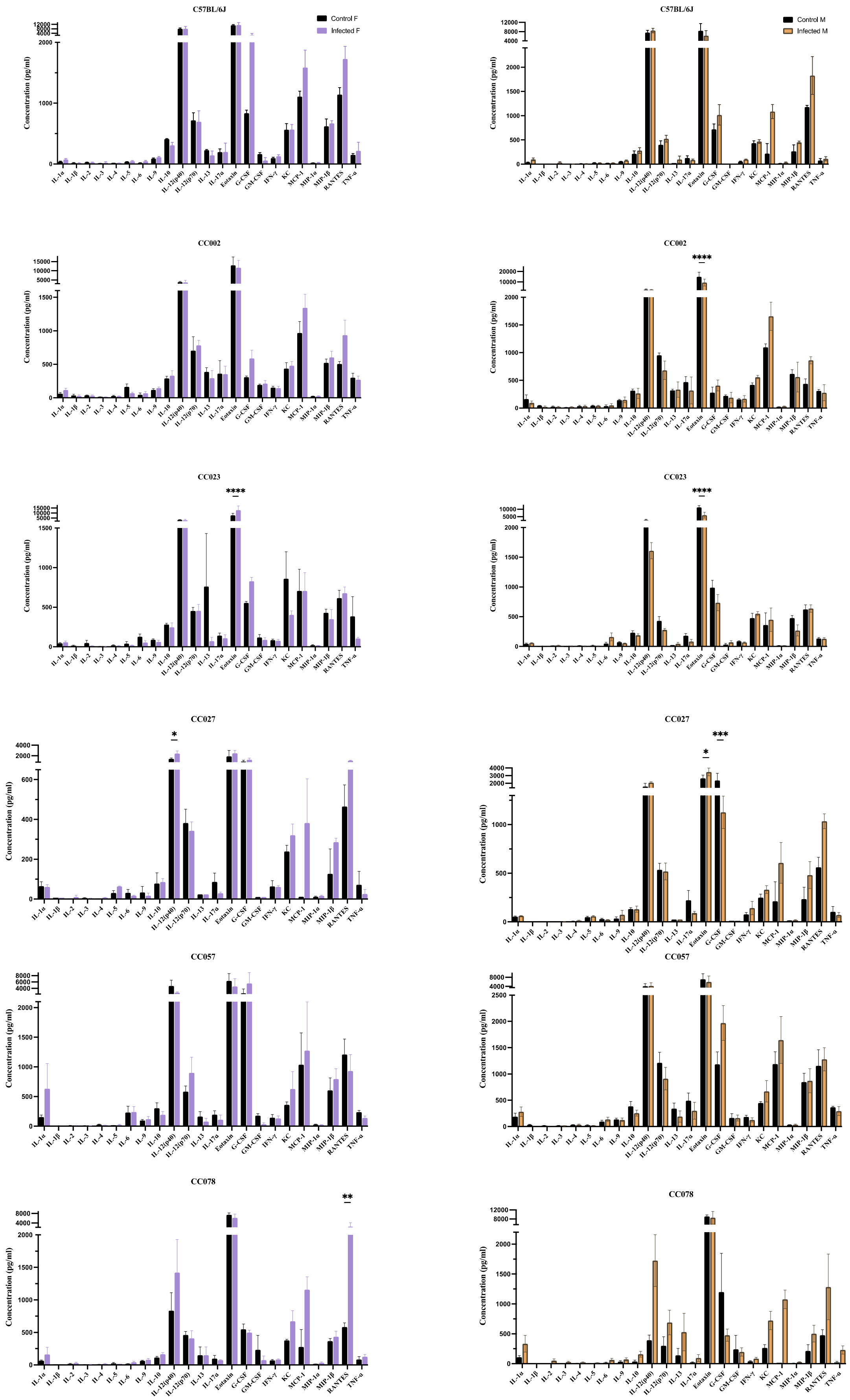
C57BL/6J
Control F
Infected F
Control M
Infected M
CC002
CC023
CC027
CC057
CC078
Concentration (pg/ml)
****
*
***
**
IL-1α
IL-1β
IL-2
IL-3
IL-4
IL-5
IL-6
IL-9
IL-10
IL-12(p40)
IL-12(p70)
IL-13
IL-17α
Eotaxin
G-CSF
GM-CSF
IFN-γ
KC
MCP-1
MIP-1α
MIP-1β
RANTES
TNF-α

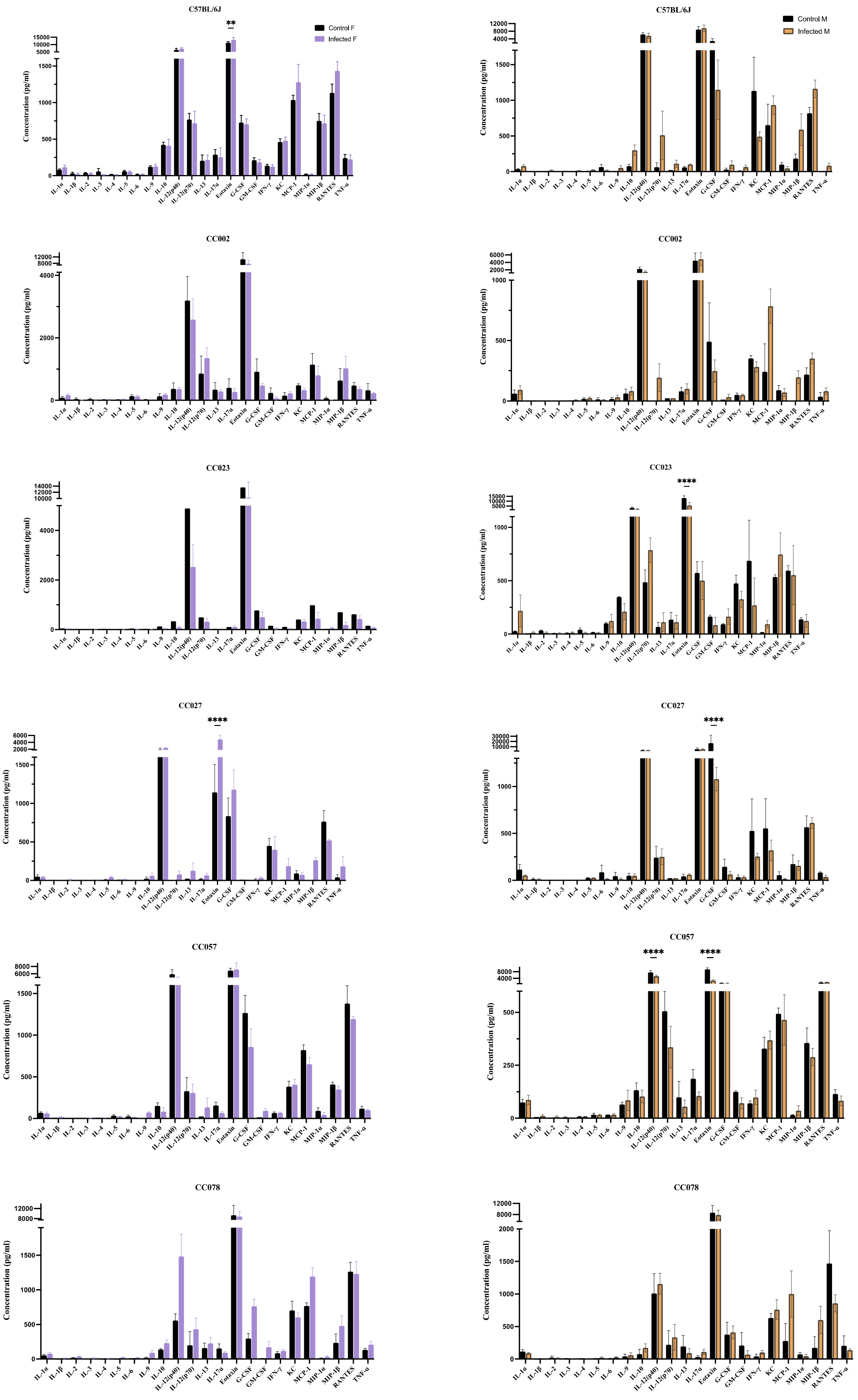

Figure S3. 4 d.p.i. Cyto/chemokine sex differences between control and infected groups. *p<0.05, **p<0.01, ***p<0.001, ****p<0.0001.
